# Local Versus Global Effects of Isoflurane Anesthesia on Visual Processing in the Fly Brain

**DOI:** 10.1523/ENEURO.0116-16.2016

**Published:** 2016-08-01

**Authors:** Dror Cohen, Oressia H. Zalucki, Bruno van Swinderen, Naotsugu Tsuchiya

**Affiliations:** 1 School of Psychological Sciences, Monash University, Melbourne 3168, Victoria, Australia; 2 Queensland Brain Institute, The University of Queensland, Brisbane 4072, Queensland, Australia; 3 Monash Institute of Cognitive and Clinical Neuroscience, Monash University, Melbourne 3168, Victoria, Australia

**Keywords:** anesthesia, consciousness, drosophila, frequency tagging, isoflurane, SSVEP

## Abstract

What characteristics of neural activity distinguish the awake and anesthetized brain? Drugs such as isoflurane abolish behavioral responsiveness in all animals, implying evolutionarily conserved mechanisms. However, it is unclear whether this conservation is reflected at the level of neural activity. Studies in humans have shown that anesthesia is characterized by spatially distinct spectral and coherence signatures that have also been implicated in the global impairment of cortical communication. We questioned whether anesthesia has similar effects on global and local neural processing in one of the smallest brains, that of the fruit fly (*Drosophila melanogaster*). Using a recently developed multielectrode technique, we recorded local field potentials from different areas of the fly brain simultaneously, while manipulating the concentration of isoflurane. Flickering visual stimuli (‘frequency tags’) allowed us to track evoked responses in the frequency domain and measure the effects of isoflurane throughout the brain. We found that isoflurane reduced power and coherence at the tagging frequency (13 or 17 Hz) in central brain regions. Unexpectedly, isoflurane increased power and coherence at twice the tag frequency (26 or 34 Hz) in the optic lobes of the fly, but only for specific stimulus configurations. By modeling the periodic responses, we show that the increase in power in peripheral areas can be attributed to local neuroanatomy. We further show that the effects on coherence can be explained by impacted signal-to-noise ratios. Together, our results show that general anesthesia has distinct local and global effects on neuronal processing in the fruit fly brain.

## Significance Statement

Understanding the neural basis of general anesthesia is important for both clinical and consciousness research. Studies in humans show that general anesthesia has distinct local and global effects. Here, we show homologous findings in the fruit fly brains, taking us a step closer to understanding how the loss of consciousness under general anesthesia is evolutionarily conserved across different neuroanatomies. Our unique combination of methods demonstrates that (1) frequency tagging can be used to dissect the neural mechanisms of general anesthesia, (2) anesthesia manipulations deepen our mechanistic understanding of neural processing, and (3) simple modeling can help to clarify unexpected results.

## Introduction

Volatile general anesthetics, such as isoflurane, abolish behavioral responsiveness in all animals, but the neural underpinnings of this phenomenon remain unclear (van Swinderen and Kottler, 2014). Although the cellular and molecular mechanisms through which general anesthetics work have been quite well characterized (Franks, 2008; Garcia et al., 2010; Brown et al., 2011), it is unclear what aspect of neural activity is at the core of the profound disconnection from the environment that is induced by all general anesthetics. The difficulty in understanding the mechanisms of general anesthesia may be attributed in part to these drugs targeting multiple processes, from sleep circuits to the synaptic release machinery (van Swinderen and Kottler, 2014).

In humans, general anesthetics have several stereotypical effects on neural activity, as measured by the electroencephalogram (EEG), the best known of which is the increase in delta (0.5-4 Hz) power that is associated with alternation between highly coordinated UP (depolarized) and DOWN (hyperpolarized) states, which is also observed during human non-rapid eye movement (REM) sleep (Murphy et al., 2011; Lewis et al., 2012). Human EEG studies using propofol anesthesia also show an increase in coherent frontal oscillations in the alpha band (8-12 Hz), a potential mechanism for impaired cortical communication (Cimenser et al., 2011; Supp et al., 2011). Human studies combining transcranial magnetic stimulation (TMS) with EEG show that midazolam, propofol, and xenon dramatically disrupt corticocortical communication in response to a TMS pulse (Ferrarelli et al., 2010; Sarasso et al., 2015). These are in agreement with the theoretical suggestion that anesthetics cause the loss of consciousness by interrupting the global integration of cortical activity (Alkire et al., 2008).

The fly model offers a unique opportunity for studying anesthetic action, because it offers the smallest brain (∼100,000 neurons) that is potentially affected by general anesthetics in the same way as the human brain. Isoflurane anesthesia abolishes behavioral responsiveness in fruit flies (Kottler et al., 2013; Zalucki et al., 2015a, Zalucki et al., 2015b), and this is associated with decreased brain activity (van Swinderen, 2006). Genetic manipulations in *Drosophila melanogaster* are shedding new light on anesthetic action, suggesting that general anesthesia might also involve presynaptic mechanisms as well as the potentiation of sleep circuits (van Swinderen and Kottler, 2014). However, it is currently unclear whether the effects of general anesthesia on neural processing are conserved across all brains, regardless of specific neuroanatomy. To investigate this, we recorded neural activity from multiple regions of the fly brain simultaneously during wakefulness and isoflurane anesthesia, while also measuring brain and behavioral responses to exogenous stimuli.

We used a recently developed multielectrode preparation (Paulk et al., 2013) to record evoked local field potentials (LFPs) across the fly brain in response to flickering visual stimuli. The flickering stimuli produced a periodic response, known as steady-state visually evoked potentials (SSVEPs; Norcia et al., 2015), that allowed us to accurately track the responses in the frequency domain across brain structures, from the optic lobes to the central brain. We hypothesized that isoflurane would globally reduce the power of the SSVEP throughout the brain, but that impaired signal transmission would have a greater effect in the central brain compared with the optic lobes. We found that isoflurane indeed reduced SSVEP power and coherence in central brain areas, but that, surprisingly, responses in the periphery actually increased under isoflurane exposure. We explain these results using a simple model based on known fly neuroanatomy, which suggests a possibility that isoflurane induces an imbalance of the On and Off visual pathways. We further show that the relationship between SSVEP power and coherence can be explained by explicitly considering the relationship between evoked responses and spontaneous brain activity. These results suggest that volatile anesthetics have distinct local and global level effects in all brains, regardless of their specific neuroanatomy, but also that local neuroanatomy is key to understanding anesthetic effects.

## Materials and Methods

### Animals

**Figure 1.**
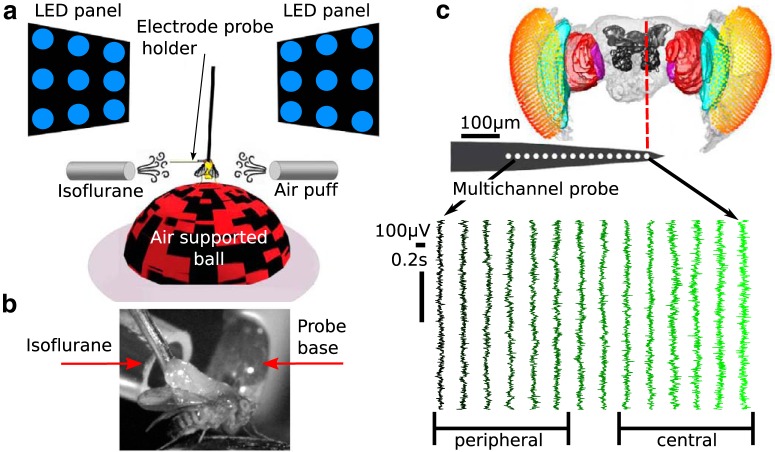
Experimental procedure and paradigm. ***a***, Experimental setup. Flies were dorsally fixed to a tungsten rod and placed on an air-supported ball, where they could freely walk. Flickering stimuli at 13 or 17 Hz were presented through two LED screens to the left and right. Isoflurane in different volumetric concentrations was delivered through a rubber hose. An air puff was used as a startle stimulus to gauge the responsiveness of the flies. A 16-contact electrode probe mounted on an electrode holder was inserted laterally from the left. Only the electrode holder is visible at the depicted scale. ***b***, A close-up view contralateral to the insertion site showing the fly, isoflurane delivery hose, and probe base. ***c***, Example of spontaneous (no presentation of visual stimuli), bipolar rereferenced data before anesthesia (0% isoflurane) from a half-brain probe recording (see Electrode probe insertion). A standardized fly brain is shown for comparison (Paulk et al., 2013, 2015). The electrode contacts are indicated by white dots (not to scale). Channels are grouped as peripheral, estimated to correspond to the optic lobe, and central, estimated to correspond to the central brain.

Female laboratory-reared *D. melanogaster* (Canton S wild type) flies (3–7 d post eclosion) were collected under cold anesthesia and positioned for tethering. Flies were dorsally glued to a tungsten rod using dental cement (Synergy D6 FLOW A3.5/B3, Coltène Whaledent), which was cured with blue light. Dental cement was applied to the neck to stabilize the head. The wings of the flies were glued to the tungsten rod to prevent wingbeats or attempted flight during recording. Tethered flies were positioned above a 45.5 mg air-supported Styrofoam ball (Fig. 1*a*,*b*), similar to that described by Paulk et al. (2013).

### Electrode probe insertion

Linear silicon probes with 16 electrodes (Neuronexus Technologies) were inserted laterally to the eye of the fly, and perpendicularly to the curvature of the eye. Insertion was performed with the aid of a micromanipulator (Merzhauser), with the electrode recording sites facing posteriorly. For the majority of experiments (14 flies), probes with electrode site separation of 25 μm (3mm-25-177) and 375 μm from base to tip (Fig. 1*c*) were used. This probe covers approximately half of the brain and henceforth is referred to as the “half-brain probe.” In two additional flies, a probe of 3mm-50-177, with electrode site separation of 50 μm and measuring 703 μm from base to tip was used. This probe covers approximately the whole brain and is referred to as the “whole-brain probe.” Probe tip width (33 μm), base width (123 μm), thickness (15 μm), and electrode site area (177 μm^2^) are identical for both probes.

A sharpened fine tungsten wire (0.01 inch diameter, A-M Systems) acted as the reference electrode and was placed superficially in the thorax. Recordings were made using a Tucker-Davis Technologies multichannel data acquisition system with a sampling rate of 25 kHz.

The probes were fully inserted until all electrode sites were recording neural activity, which was confirmed by the presentation of visually flickering stimuli (1 and 13 Hz; see Visual stimuli) and observing SSVEPs at the most peripheral electrode site (furthest from the probe tip). The probe was then gently retracted until the most peripheral site showed little to no neural activity. We assumed that this indicated that the most peripheral site was placed just outside the eye. This ensured consistent probe insertion depth among flies.

### Visual stimuli

Flickering blue lights (spectral peak at 470 nm with a 30 nm half-peak width) were presented through two LED panels (Fig. 1*a*). The panels were flickered on and off (square wave, 50% duty cycle) at 13.4 Hz (hereafter cited as 13 Hz) or 16.6 Hz (hereafter cited as 17 Hz), and either to the left or to the right of the fly. There were thus eight possible flicker configurations. These are [off 13], [off 17], [13 off], [17 off], [13 13], [13 17], [17 13], and [17 17], where the number represents the flicker frequency and the location represents the left or right LED panel. An “off” signifies that we turned off the blue LED lights in the respective panel. Visual flickers were presented in sets of 80 trials, consisting of 10 presentation of each flicker configuration. A trial lasted 2.3 s, and the intertrial interval was 0.8 s, taking 248 s to complete the 80 trials. The flicker configuration order was randomly generated with the added restriction that consecutive trials consisted of different flicker configurations. Panel voltage levels were recorded at 25 kHz in the same recording system as the electrophysiological signals. The LED lights were turned off except during the period of visual stimulation (see Fig. 4*a*).

In three of the flies (two with the whole-brain probe and one with the half-brain probe, all with the graded anesthesia manipulation; see Isoflurane delivery), we also included an additional flicker configuration of [1 1] (1 Hz flicker in both panels). This stimulus was presented once for 20 s before the start of the 80 trials described above. These three flies are used for evaluating the modeled SSVEPs (see Modeling the SSVEPs).

### Grouping flicker configurations as ipsilateral or contralateral

The eight flicker configurations were chosen to isolate the effects of flicker frequency (13 vs 17 Hz), flicker interaction (e.g., [13 off] vs [13 17]), and flicker location (e.g., [13 off] vs [off 13]). However, we found that grouping trials as either ipsilateral or contralateral simplified the results and was sufficient to substantiate all our claims (see Results). Under this classification scheme, trials in which a single flicker was presented at the left panel were labeled ipsilateral (see Fig. 3*a*,*b*, [13 off] and [17 off]). Trials in which a single flicker was presented at the right panel were labeled contralateral (see Fig. 3*a*,*b*, [off 13] and [off 17]). Because the ipsilateral flicker dominated the response, we classified trials in which the same flicker was presented in both panels as ipsilateral (see Fig. 3*a*,*b*), and trials in which different flickers were presented in both panels as ipsilateral at the frequency of the ipsilateral panel and contralateral at the frequency of the contralateral panel (see Fig. 3*a*,*b*).

### Isoflurane delivery

Isoflurane was delivered onto the fly through a rubber hose connected to an evaporator (Mediquip; Fig. 1*a*,*b*). The isoflurane was blown onto the fly at a constant flow of 2l/min and continuously vacuumed from the opposite side of the fly. Following the gas chromatography procedure described by Kottler et al. (2013) for measuring isoflurane concentration, we found that the actual concentration near the fly body was 0.3% (vol) when the concentration at the evaporator was set to 1%. Throughout this article, we report isoflurane concentration as the linearly estimated concentration at the fly body, not at the evaporator.

Isoflurane concentrations were manipulated in either a graded or a binary manner over the blocks. In the graded manipulation (*N* = 3 with the half-brain probe and *N* = 2 with the whole-brain probe), concentrations were incrementally and sequentially increased over five levels and then reduced to 0%; 0% (air) → 0.06% → 0.18% → 0.3% → 0.45% → 0.6% → 0% (recovery). In the binary manipulation (*N* = 10 with the half-brain probe), the isoflurane concentration was manipulated over three blocks [0% (air) → 0.6% → 0% (recovery)]. Throughout the article, we distinguish the two periods of 0% isoflurane as 0% (air) and 0% (recovery), before and after drug exposure, respectively. In one fly in which we used the half-brain probe, we administered the graded manipulation up to 0.45%. In a subset of the flies (*N* = 8 of 14 with the half-brain probe), an additional recovery block [0% (recovery 2)] was performed.

### Air puff stimuli and behavioral responsiveness

An olfactory stimulus controller (custom built) was used to deliver six air puffs to gauge the behavioral responsiveness of the flies in each concentration of isoflurane. The inter-air puff duration was ∼1.5 s. Air puffs were delivered before and after the presentation of visual stimuli (see Fig. 4*a*). Fly movement activity was recorded with a 602f-2 Firewire Camera (Basler) and a 1-6010 12× zoom lens (Navitar) at 30 frames/s, time locked to the onset of the air puff. We used the video data to assess the behavioral responsiveness of the flies under anesthesia (see Movement analysis).

### Experimental protocol

After inserting a probe and confirming the visible responses of the flies to an air puff, we initiated our experimental protocol. An experiment consisted of several blocks, each at a different concentration of isoflurane (see Fig. 4*a*). Each block started with the delivery of a series of air puffs, used to gauge the responses of the fly and to establish the depth of anesthesia (see Movement analysis). Thirty seconds after the startle stimulus, 80 trials of visual flickers were presented (see Visual stimuli). After the completion of 80 trials, the flies were left for an additional 30 s, and then a second episode of air puffs was delivered. After the last air puff was delivered, the isoflurane concentration was immediately changed. Flies were left for 180 s to adjust to the new isoflurane concentration before the next block commenced.

### Movement analysis

To confirm the depth of anesthesia, fly movements were analyzed in response to the air puffs. The recorded movies were analyzed to extract the amount of overall movement using custom software written in MATLAB (MathWorks). First, movies were downsampled to 5 frames/s and converted to grayscale. Second, individual images were annotated with the corresponding isoflurane concentration (*k* = [0 (air), 0.06, 0.18, 0.3, 0.45, 0.6, 0 (recovery), 0 (recovery 2)]%) and saved. Third, images were cropped to include only the body of the fly and were tailored for each fly. Fourth, the mean square error (MSE) between consecutive images across all pixels was calculated, giving one MSE value for each pair of consecutive frames at each isoflurane concentration, *k*%;

$$M_{MSE}^{k}(i)=\frac{1}{N}\sum_{x}\sum_{y}{\left(image_{i+1}^{k}(x,y)-image_{i}^{k}(x,y)\right)}^{2},$$

where *N* is the total number of pixels in each image, $image_{i}^{k}(x,y)$ and $image_{i+1}^{k}(x,y)$ represent the grayscale value of pixel (*x*,*y*) in frame *i* and *i + 1*, respectively, and the sum is taken over all pixels in the image. Finally, the resulting values were averaged over the two episodes of six air puffs in each block with isoflurane concentration *k* (∼90 frames in total; see Fig. 4*a*) to obtain ${\bar{M}}_{MSE}^{k}$. For comparison across flies, we further normalized the values for each fly by dividing the value in *k*% isoflurane (= ${\bar{M}}_{MSE}^{k}$) by the value in 0% isoflurane (air; = ${\bar{M}}_{MSE}^{0}$). We refer to the resulting quantity as the movement index (MI^k^). MI values above and below 1.0 indicate increased and decreased movement compared with 0% isoflurane, respectively. When computing the MI for the recovery period, we used the images from the last experimental block of each fly, which was 0% (recovery) for eight flies and 0% (recovery 2) for five flies.

### Local field potential analysis

Electrophysiological data was recorded at 25 kHz and downsampled to 1000 Hz for all subsequent analyses. The most peripheral electrode site was removed from the analysis as it was outside the brain (see Electrode probe insertion). The remaining 15 electrodes sites were bipolar rereferenced by subtracting neighboring electrodes to obtain a set of 14 differential signals, which we refer to as “channels” hereafter (Fig. 1*c*).

For SSVEP analysis, we segmented the data into 2.3 s epochs according to the flicker configuration and isoflurane concentration. We removed line noise at 50 Hz using the *rmlinesmovingwinc.m* function from the Chronux toolbox (http://chronux.org/; Mitra and Bokil, 2007) with three tapers, a window size of 0.7 s, and a step size of 0.35 s.

#### Analyzing power

For each fly, we denote the power of the LFP during visual stimulation at frequency *f*, in channel *i* (1–14), flicker configuration *l* (1–8), and isoflurane concentration *k*% ([0 (air), 0.06, 0.18, 0.3, 0.45, 0.6, 0 (recovery)]) as $S_{E}^{ilk}(f)$, with subscript *E* meaning “evoked.” $S_{E}^{ilk}(f)$ is in units of 10log10 (μV^2^), averaged (in the log scale) over the 10 repetitions of the flicker configuration (see Visual stimuli; see Fig. 4*a*). $S_{E}^{ilk}(f)$ was calculated over the 2.3 s trial period using the multitaper method (*mtspectrumc.m*, http://chronux.org/; Mitra and Bokil, 2007) with three tapers, giving a half bandwidth of ∼0.87 Hz (Mitra and Pesaran, 1999), which is sufficiently fine for our claims in this article. We denote spontaneous power at frequency *f* in channel *i*. and isoflurane *k*% as $S_{S}^{ik}(f)$, with subscript *S* meaning “spontaneous.” $S_{S}^{ik}(f)$ is the power averaged across four 2.3-s-long segments before the start of the visual flicker presentation in units of 10log10 (μV^2^; see Fig. 4*a*).

When presenting the results for *k* = 0% (air), we corrected for baseline levels by subtracting the spontaneous power from SSVEP power, as follows:

(1.1) $$S_{EB}^{il}(f)=S_{E}^{il0}(f)-S_{S}^{i0}(f),$$

(subscript B for baseline correction). $S_{EB}^{il}(f)$ is reported in decibels, emphasizing that the subtraction is performed after conversion to the log scale.

We use the symbols *f*_1_ and *f*_2_ to refer to the tag frequency or twice the tag frequency, respectively. The frequencies corresponding to *f*_1_ and *f*_2_ are flicker configuration dependent (e.g., when the flicker configuration was [13 13], *f*_1_ = 13 Hz and *f*_2_ = 26 Hz). We refer to power at frequency *f_n_* as the average power from −0.5 to +0.5 around the frequency of interest, as follows:

(1.2) $$S_{E}^{ilk}(f_{n})=\frac{1}{N}\sum_{f_{n}-0.5}^{f_{n}+0.5}S_{E}^{ilk}(f)$$

where *N* = 4 is the number of frequency bins over which the sum is evaluated. The baseline-corrected SSVEP power $S_{EB}^{il}(f_{n})$ was obtained by the substitution of $S_{EB}^{il}(f)$ for $S_{E}^{ilk}(f)$ in Equation 1.2.

When reporting SSVEP power for ipsilateral and contralateral flicker configurations, we separately averaged the flicker configurations for each grouping and the corresponding tags (see Grouping flicker configurations as ipsilateral or contralateral; see Fig. 3*a*,*b*). For example, $S_{E}^{ik\;Ipsi}(f_{1})$ refers to the average power at *f*_1_ across the six flicker configurations where the flicker was presented ipsilateral to the probe insertion site (13 Hz for [13 off], [13 13], and [13 17], and 17 Hz for [17 off], [17 17], and [17 13]). The baseline-corrected SSVEP power $S_{EB}^{ik\;Ipsi}(f_{n})$ was obtained by substituting $S_{EB}^{il}(f)$ for $S_{E}^{ilk}(f)$ and repeating the derivation.

The effect of *k*% isoflurane on SSVEP power is denoted by the symbol Δ and obtained by subtracting respective values in 0% (air) isoflurane, as follows:

(1.3) $$\begin{array}{l} \Delta S_{E}^{ilk}(f)=S_{E}^{ilk}(f)-S_{E}^{il0}(f)\\ \Delta S_{S}^{ik}(f)=S_{S}^{ik}(f)-S_{S}^{i0}(f) \end{array}$$

When reporting the effect on power at the tagged frequency (*f* = *f*_1_ or *f*_2_), we averaged the power around the tagged frequency, as in Equation 1.2. To obtain the average for ipsilateral/contralateral flicker configurations ($\Delta S_{E}^{ik\;Ipsi/Contra}(f_{1/2})$), we repeated the derivation above with $\Delta S_{E}^{ilk}(f_{1/2})$ substituted for $S_{E}^{ilk}(f_{1/2})$.

#### Analyzing coherence

We analyzed coherence between channel pairs using the function *coherencyc.m* in the Chronux toolbox (Mitra and Bokil, 2007) with five tapers, giving a half bandwidth of 1.40 Hz (Mitra and Pesaran, 1999), which is sufficient for our claims. Our notation and terminology for coherence parallel those used for reporting power, as described below.

SSVEP coherence for channel pair (*i*,*j*), flicker configuration *l*, and isoflurane concentration *k%*, $C_{E}^{ijlk}(f)$, is calculated for the SSVEPs over the 2.3 s trials and averaged over the 10 repetitions of the flicker configuration. As spontaneous coherence $C_{S}^{ijlk}(f)$, we report coherence averaged across four 2.3-s-long segments before the start of visual flicker presentation (see Fig. 4*a*).

Baseline-corrected SSVEP coherence is used when presenting results in 0% (air) isoflurane and defined as follows:

(2.1) $$C_{EB}^{ijl}(f)=C_{E}^{ijl0}(f)-C_{S}^{ij0}(f).$$

As for power, we refer to coherence at frequency *f_n_* ($C_{EB}^{ijl}(f_{n}),\;C_{E}^{ijl}(f_{n})$) as the average coherence from −0.5 to +0.5 Hz around the tagged frequency.

We calculated SSVEP and spontaneous coherence between all channel pairs, resulting in 91 (14*13/2) unique values at every frequency. To summarize these data in a concise way, we grouped channel pairs into periphery (channels 1–6) and center (channels 9–14; Fig. 1*c*). We report periphery (P), center-periphery (CP), and center (C) coherence as averaged across all pairs of the electrodes within periphery, between center and periphery, and within center, respectively;

(2.2) $$\begin{array}{l} {\text{C}}_{\text{E}}^{\text{Plk}}\left(f_{n}\right)=\frac{1}{15}\overset{6}{\underset{j=i+1}{\sum }}\overset{5}{\underset{i=1}{\sum }}{\text{C}}_{\text{E}}^{\text{ijlk}}\left(f_{n}\right)\\ {\text{C}}_{\text{E}}^{\text{CPlk}}\left(f_{n}\right)=\frac{1}{36}\overset{6}{\underset{j=1}{\sum }}\overset{14}{\underset{i=9}{\sum }}{\text{C}}_{\text{E}}^{\text{ijlk}}\left(f_{n}\right)\\ {\text{C}}_{\text{E}}^{\text{Clk}}\left(f_{n}\right)=\frac{1}{15}\overset{14}{\underset{j=i+1}{\sum }}\overset{13}{\underset{i=9}{\sum }}{\text{C}}_{\text{E}}^{\text{ijlk}}\left(f_{n}\right) \end{array}$$

where the superscripts P, CP, and C replace the channel pair superscript. The upper and lower limits of the sums reflect the grouping into peripheral (1–6) and center (9–14) channels, and take into account the fact that coherence is invariant with respect to channel order (C*^ij^* = C*^ji^*), while excluding coherence between a channel and itself (C*^ii^* = 1). Our results were not sensitive to the exact grouping, such that other schemes, for example, periphery = channels 2–5, center = channels 10–13, gave similar results. We obtained the analogous quantities for baseline-corrected SSVEP coherence at *f_n,_* $C_{EB}^{Pl}(f_{n})$, $C_{EB}^{CPl}(f_{n})$, and $C_{EB}^{Cl}(f_{n})$ by substitution of $C_{EB}^{ijl}(f_{n})$ for $C_{E}^{ijlk}(f_{n})$ in Equation 2.2.

Paralleling the power analysis, we report SSVEP coherence for ipsilateral and contralateral configurations at *f*_1_ and *f_2_* [e.g., ${\text{C}}_{\text{E}}^{\text{Pk}\;\text{Ipsi}}\left(f_{1}\right)$ refers to the average coherence at *f*_1_ across six flicker conditions, where the flicker was presented ipsilateral to the probe insertion site (see Fig. 3*a*,*b*)]. The baseline-corrected SSVEP coherence [e.g., ${\text{C}}_{\text{EB}}^{\text{Pk}\;\text{Ipsi}}\left(f_{1}\right)$] and the effect of *k*% isoflurane [e.g.,$\Delta {\text{C}}_{\text{E}}^{\text{Pk}\;\text{Ipsi}}\left(f_{1}\right)$] were defined similarly to the analogous quantities for power [e.g., ${\text{S}}_{\text{EB}}^{\text{ik}\;\text{Ipsi}}\left(f_{1}\right)$ and $\Delta {\text{S}}_{\text{E}}^{\text{ik Ipsi}}\left(f_{1}\right)$].

### Modeling the SSVEPs

We modeled the SSVEPs as the sum of two separate linear responses corresponding to the On and Off pathways (see Fig. 5*a*). The input (depicted as a square wave) is differentiated to extract points of luminance increments and decrements before splitting into two streams corresponding to the On and Off pathways. The responses of the On and Off pathways are summed to give the modeled SSVEP. Mathematically, the output of the model is given by the following:

(3.1) $$\begin{array}{ccc} v\left(t\right) & = & h_{on}⊗u_{on}+h_{off}⊗u_{off} \end{array},$$

where v(t) is the output voltage, $h_{on}$ and $h_{off}$ are the impulse responses of the On and Off pathways, respectively, and ⊗ denotes convolution in the time domain. *u_on_* and *u_off_* are the half-wave rectified inputs to the On and Off pathways, such that *u*_on_(*t*) = 1 and *u*_off_(*t*) = 1 signify an increase and a decrease in luminance at time *t*, respectively. Note that beyond the rectification nonlinearity the model is a linear multiple-input/single-output model (Bendat and Piersol, 2000). Our model assumes that the effect of isoflurane on the SSVEPs can be explained by changes to the impulse responses $h_{on}$ and $h_{off}$ alone.

We note that the nonlinearity is cancelled if the impulse response of the On pathway is identical and opposite to the impulse response of the Off pathway (Regan and Regan, 1988). Subbing $h_{on}=-h_{off}$ into Equation 3.1 and using $u\prime \left(t\right)=u_{on}\left(t\right)-u_{off}\left(t\right)$, we obtain the following:

(3.2) $$\begin{array}{ccc} v\left(t\right) & = & h_{on}⊗u\prime \left(t\right) \end{array}.$$

Because u′(t) is simply the (linearly) differentiated input (u′(t)=u(t)−u(t−1)), Equation 3.2 shows that the model reduces to a single linear operation of the (linearly differentiated) input when $h_{on}=-h_{off}$.

The frequency response of the model is given by the following:

$$\begin{array}{ccc} V\left(f\right) & = & H_{on}\left(f\right)U_{on}\left(f\right)+H_{off}\left(f\right)U_{off} \end{array}\left(f\right),$$

where the convolution in Equation 3.1 is replaced by multiplication, and capital letters represent the Fourier transforms of their respective variables. The power spectrum of the response of the model is given by the following:

(3.3) $$V\left(f\right)V{\left(f\right)}^{*}=V_{on}\left(f\right)V_{on}{\left(f\right)}^{*}+V_{off}\left(f\right)V_{off}{\left(f\right)}^{*}+2Re\left(V_{on}\left(f\right)V_{off}{\left(f\right)}^{*}\right),$$

where we used $V_{on}\left(f\right)=H_{on}\left(f\right)U_{on}\left(f\right)$ and $V_{off}\left(f\right)=H_{off}\left(f\right)U_{off}\left(f\right)$ for the responses of the On and Off pathways to their respective inputs. The symbol * represents conjugation, and *Re()* denotes taking the real part.

We note two things about Equation 3.3. First, the response at frequency *f* is only a function of the responses of the On and Off pathways at frequency *f*; there is no contribution from other frequencies. In the context of our experiments, this means that the prediction of the model for SSVEP power at *f*_1_ and *f*_2_ depends only on the stimulus, and the properties of the transfer functions ($H_{on/off}\left(f\right)$) at *f_1_* and *f_2_*.

Second, the prediction of the model for SSVEP power depends on the SSVEP power of the On ($V_{on}\left(f\right)V_{on}{\left(f\right)}^{*})$ and Off pathways ($V_{off}\left(f\right)V_{off}{\left(f\right)}^{*})$, but also on the cross-spectrum between the responses ($2Re\left(V_{on}\left(f\right)V_{off}{\left(f\right)}^{*}\right)$).

The On and Off impulse responses at each channel *i* and isoflurane concentration *k* $h_{on/off}^{ik}$ were estimated by averaging the LFP over 20 on–off cycles of the [1 1] flicker configuration, which we presented to three flies (see Visual stimuli; see Fig. 5*b*). Because the input is a square wave, the half-wave rectification effectively transforms the input into two pulse trains (see Fig. 5*a*). We then used the estimated impulse responses at each channel and the isoflurane concentration together with Equation 3.1 to predict SSVEPs for the [13 13] and [17 17] flicker configurations by setting the input to a 50% duty cycle square wave with periods (1/13 Hz) and (1/17 Hz), respectively. By computing the Fourier transform of the modeled SSVEPs at each channel *i*, the two flicker configurations *l* ([13 13] and [17 17]) and isoflurane concentration *k*, we obtained the prediction of the model for SSVEP power ${\hat{\text{ S}}}_{\text{E}}^{\text{ilk}}\left(f\right)$. The prediction of the model for the effect of *k*% isoflurane ${\Delta \hat{\text{S}}}_{\text{E}}^{\text{ilk}}\left(f\right)$ is obtained by substituting ${\hat{\text{S}}}_{\text{E}}^{\text{ilk}}\left(f\right)$ for the measured SSVEP power (${\text{S}}_{\text{E}}^{\text{ilk}}\left(f\right)$) in Equation 1.3. The quantities ${\Delta \hat{\text{S}}}_{\text{E}}^{\text{ilk}}\left(f_{n}\right)$ and ${\hat{\text{S}}}_{\text{E}}^{\text{ilk}}\left(f_{n}\right)$ were obtained by averaging from −0.5 to +0.5 Hz around the frequency of interest. Note that the model does not have any degrees of freedom for fitting, once the impulse responses are determined by the simple averaging of the data over 20 on–off cycles of the [1 1] flicker configuration. Nothing further is estimated or fit from the data.

### Evaluating the SSVEP model

To investigate the relationship between the model and the data, we performed linear regression between the model-predicted and observed effects of isoflurane on SSVEP power, as follows:

$${\text{ΔS}}_{\text{E}}^{\text{ilk}}\left(f_{1/2}\right)=b{\Delta \hat{\text{S}}}_{\text{E}}^{\text{ilk}}\left(f_{1/2}\right)+c,$$

where *b* and *c* were estimated in R (https://www.r-project.org/; R Core Development Team, 2015) using the *lm* function. We report the Pearson’s correlation coefficient ρ between the prediction of the model and the observed data, and 95% confidence intervals on the slope (*b*) and intercept (*c*) obtained by the *confint* function. Note that a perfect fit between model and data is given by ρ=1 and the line (*b* = 1, *c* = 0).

We performed the regression over three flies, all channels (1–14), two flicker configurations ([13 13] and [17 17]), and both *f*_1_ and *f*_2_, giving 168 paired data points in total. We used the highest concentration of isoflurane [represented as the superscript *H* in ΔS^H^_E_ (see Fig. 5*g*)] presented to each fly, where *k* = 0.6% for two flies and *k* = 0.45% for one fly (see Isoflurane delivery).

### Signal-to-noise ratio-based estimation of coherence

To investigate the relationship between evoked power and coherence, we assumed a linear framework in which the SSVEPs for each channel pair are related through a linear transfer function in the presence of noise (see Fig. 6*b*). This framework is conceptually related to the SSVEP model but is completely independent in its implementation and evaluation.

In this framework, the SSVEP at channel *i*, given by *v_i_*(*t*) passes through the linear transfer function *H_ij_*(*f*) to give the SSVEP at channel *j*, *v_j_*(*t*). Independent noise enters at each channel separately [*n_i_*(*t*) and *n_j_*(*t*)] to give the recorded responses *y_i_*(*t*) and *y_j_*(*t*). Under these assumptions, squared coherence between channel pairs has an analytical description (for the detailed derivation, see Bendat and Piersol, 2000), as follows:

$$\frac{1}{C^{ij}{\left(f\right)}^{2}}=1+\frac{S_{N}^{i}\left(f\right)}{S_{V}^{i}\left(f\right)}+\frac{S_{N}^{j}\left(f\right)}{S_{V}^{j}\left(f\right)}+\frac{S_{N}^{i}\left(f\right)S_{N}^{j}\left(f\right)}{S_{V}^{i}\left(f\right)S_{V}^{j}\left(f\right)},$$

where $S_{N}^{i},S_{N}^{j},S_{V}^{i}$, and $S_{V}^{j}$ are the power spectrums of *n_i_*, *n_j_*, *v_i_*, and *v_j_*, respectively. If we define the signal-to-noise ratios (SNRs) as $\frac{1}{SNR^{i}\left(f\right)}=\frac{S_{N}^{i}\left(f\right)}{S_{V}^{i}\left(f\right)}$ and $\frac{1}{SNR^{j}\left(f\right)}=\frac{S_{N}^{j}\left(f\right)}{S_{V}^{j}\left(f\right)}$, then:

(4.1) $$\frac{1}{C^{ij}{\left(f\right)}^{2}}=1+\frac{SNR^{i}\left(f\right)+SNR^{j}\left(f\right)+1}{SNR^{i}\left(f\right)SNR^{j}\left(f\right)}.$$

Thus, in this simplified setting coherence is totally determined by the SNRs at the respective channels.

To evaluate the SNR-based coherence estimate, we quantified the SNR at each channel and used Equation 4.1 to obtain the prediction of the model of SSVEP coherence. First, we recalculated ${\text{S}}_{\text{E}}^{\text{ilk}}\left(f\right)$ using the same number of tapers used for the coherence analysis (i.e., five tapers; see Local field potential analysis). We then fitted power law noise to the observed SSVEP power ${\text{S}}_{\text{E}}^{\text{ilk}}\left(f\right)$, as follows:

(4.2) $${\text{S}}_{\text{N}}^{\text{ilk}}\left(f\right)=\frac{\alpha^{\text{ilk}}}{f^{\beta }{}^{\text{ilk}}},$$

where we excluded *f* values from −1.4 to +1.4 Hz around *f*_1_ and *f*_2_ for each flicker configuration (1.4 Hz corresponds to the half-bandwidth for the coherence measurement, see Local field potential analysis). The purpose of the fit is to estimate the level of neural activity that is not directly tagged by visual flickers. Following the convention in the SSVEP literature (Norcia et al., 2015), we considered the nontagged activity representing the level of noise (*n*(*t*) or *S*_N_(*f*)). The parameters ${\hat{\alpha }}^{\text{ilk}}$ and ${\hat{\beta }}^{\text{ilk}}$ were estimated by linear regression in the log–log scale in the range 1–50 Hz and used to define the noise spectrum.

We define the SNR at frequency *f* as the observed SSVEP power at *f* divided by the interpolated noise spectrum at *f* as follows:

(4.3) $${\text{SNR}}^{ilk}\left(f\right)=\frac{{\text{S}}_{\text{E}}^{\text{ilk}}\left(f\right)}{{\text{S}}_{\text{N}}^{\text{ilk}}\left(f\right)}.$$

An example of the estimated noise spectrums and resulting SNRs for two exemplar channels is shown in Figure 6, *c* and *d*. Figure 6*e* shows the resulting coherence estimate.

The predicted effect of *k*% isoflurane on coherence for channel pair (*i*, *j*) is obtained by subtracting the predicted coherence in 0% (air) isoflurane from the predicted coherence in *k*% isoflurane, as follows:

$${\Delta \hat{\text{C}}}_{\text{E}}^{\text{ijlk}}={\hat{\text{C}}}_{\text{E}}^{\text{ijlk}}-{\hat{\text{C}}}_{\text{E}}^{\text{ijl}0}.$$

For the further analyses, we grouped the electrode pairs into P, CP, and C, and for ipsilateral and contralateral flicker configurations separately as described before (see Fig. 6*f*).

To provide an overall measure of fit for the SNR-based estimation of coherence, we calculated the MSE between the prediction of the model and the observed effect of 0.6% isoflurane on SSVEP coherence in each fly, across all channel pairs (91), flicker configurations (8), and *f*_1_ and *f*_2_, as follows:

(4.4) MSE=1N∑l=18∑j=i+114∑i=113∑m=12{ΔC^Eijl0.6(fm)−ΔCEijl0.6(fm)}2,

where *N* is the total number of terms in the sum *N* = 91*8*2 = 1456 (for each of 13 flies).

#### Separating the contribution of “noise” and “signal” to the SNR-based estimation of coherence

The SNR-based estimation of coherence is completely determined by the SNR (Eq. 4.1), which in turn is a function of the estimated noise levels from nontagged frequency as well as the observed SSVEP power at the tagged frequency (Eq. 4.3). To isolate the contribution of the noise and SSVEP power to the coherence estimate, we defined two additional variants of SNR. In the first, for SNR_FN_, (where subscript FN is fixed noise), we fixed the noise spectrum to that fitted in 0% (air) isoflurane, as follows:

$${\text{SNR}}_{\text{FN}}^{ilk}=\frac{{\text{S}}_{\text{E}}^{\text{ilk}}}{{\text{S}}_{\text{N}}^{\text{il}0}},$$

thereby removing the influence of isoflurane on noise levels. In the second, for SNR_FE_ (where subscript FE is fixed SSVEP power), we fixed the SSVEP power to that observed in 0% (air) isoflurane, as follows:

$${\text{SNR}}_{\text{FE}}^{ilk}=\frac{{\text{S}}_{\text{E}}^{\text{il}0}}{{\text{S}}_{\text{N}}^{\text{ilk}}},$$

thereby removing the influence of isoflurane on SSVEP power. ${\text{SNR}}_{\text{FN}}^{ilk}$ and ${\text{SNR}}_{\text{FE}}^{ilk}$are used together with Equation 4.1 to obtain two additional estimates of coherence (C^FNijlk and C^FEijlk), the effects of *k*% isoflurane (ΔC^FNijlk and ΔC^FEijlk), as well as the grouped coherence over electrode pairs and flicker configurations as described before (ΔC^FN/FEPk Ipsi/Contra(f1/2), ΔC^FN/FECPk Ipsi/Contra(f1/2), and ΔC^FN/FEPk Ipsi/Contra(f1/2), presented in Fig. 6*g*,*h*). Paired *t* tests between the MSEs (Eq. 4.4; obtained for each fly separately), and between the observed effects of isoflurane on coherence and those predicted by each SNR variant were used for assessing statistical significance.

### Statistical analysis

We used R (https://www.r-project.org/, R Core Development Team, 2015) and lme4 (Bates et al., 2015) to perform linear mixed-effect analysis of the data. Throughout, the response variable is either power ${\text{S}}_{\text{E}}^{\text{ilk}}\left(f_{1/2}\right)$ or coherence ${\text{C}}_{\text{E}}^{\text{ijlk}}\left(f_{1/2}\right)$ with four factors corresponding to isoflurane, channel location, harmonic, and the fly.

As to the factors flicker configuration and response frequency, only a subset of all combinations is relevant for our claims. Specifically, when the flicker configuration was [13 13], [13 off], or [off 13] we analyzed power at *f*_1_ = 13 Hz and *f*_2_ = 26 Hz. When the flicker configuration was [17 17], [17 off], or [off 17] we analyzed power at *f*_1_ = 17 Hz and *f*_2_ = 34 Hz. When the flicker configuration was [13 17] or [17 13], we analyzed power at *f*_1_ = 13 and 17 Hz, and *f*_2_ = 26 and 34 Hz. Thus, replacing the response frequency factor, we included the factor flicker location that corresponds to the division into ipsilateral and contralateral flicker configurations (see Fig. 3*a*,*b*; see Grouping flicker configurations as ipsilateral and contralateral).

Among those factors, we focused on the crucial isoflurane-specific effects by including interactions between the isoflurane and flicker location factors, isoflurane and harmonic factors, and isoflurane and channel location factors, as well as the triple interaction among isoflurane, channel location, and harmonic factors. In addition, our results in 0% (air) isoflurane imply harmonic-dependent effects for flicker location and channel location, so we included interactions between flicker location and harmonic, and channel location and harmonic. We included random intercepts for all random effects to account for possible offsets between the levels of each factor.

To test for the effect of a given factor or interaction, we performed likelihood ratio tests between the full model described above and a reduced model without the factor or interaction in question (Bates et al., 2015). When applicable, we adjusted *p* values using the false discovery rate (Yekutieli and Benjamini, 1999).

## Results

### Evoked responses vary across the fly brain

**Figure 2.**
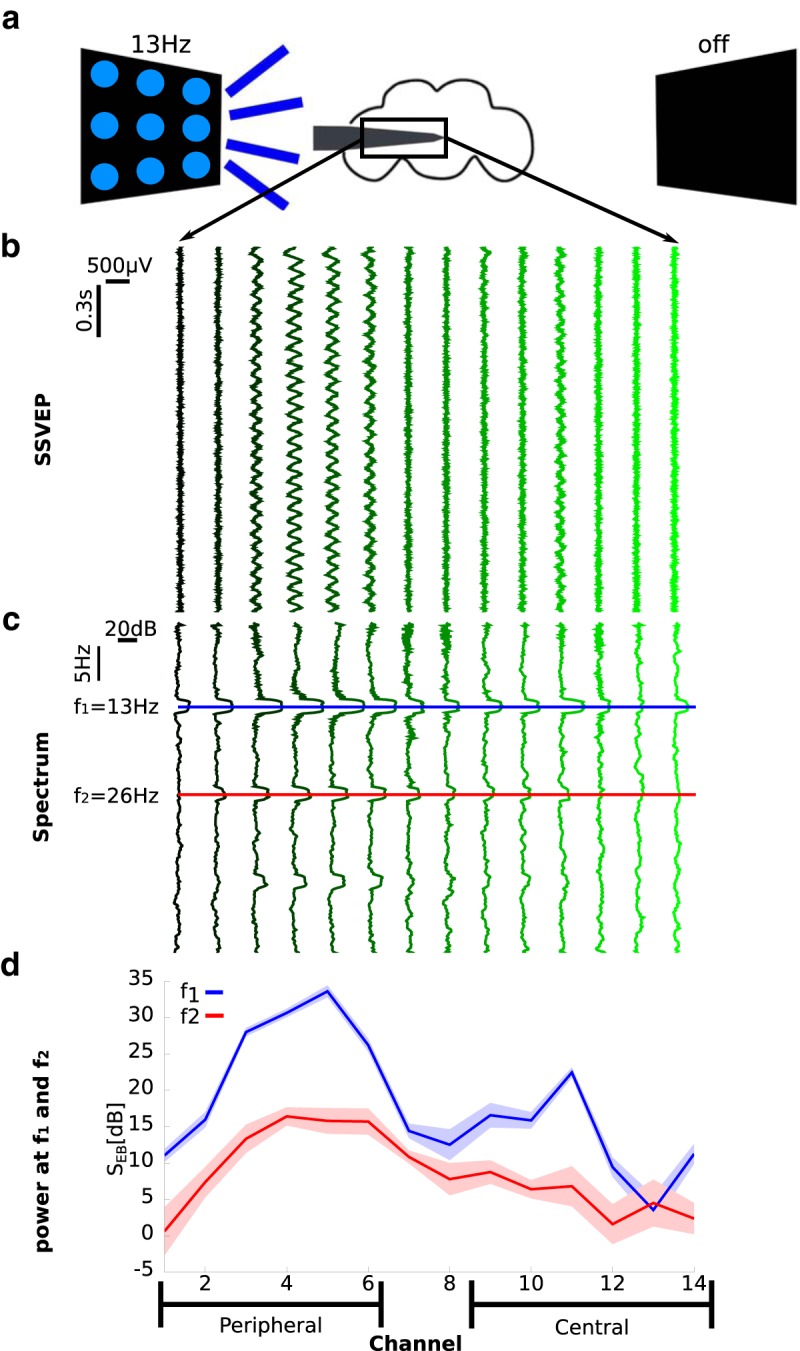
SSVEP recordings before anesthesia (0% isoflurane). ***a***, Schema of an experiment showing the electrode inserted laterally from the left. The left LED panel is shown flickering at 13 Hz, corresponding to the [13 off] flicker configuration. ***b***, Exemplar mean bipolar rereferenced SSVEP, averaged over 10 trials in the [13 off] condition. The same data from one fly are presented in ***c*** and ***d*** in different formats. ***c***, Exemplar baseline-corrected SSVEP power spectrum, averaged over the same 10 trials in ***b*** (S_EB_(*f*); see Local field potential analysis). The blue and the red lines mark the first (*f*_1_ = 13 Hz) and second (*f*_2_ = 26 Hz) harmonic, respectively. ***d***, Baseline-corrected SSVEP power at *f*_1_ (blue) and *f*_2_ (red) for the 10 trials of the [13 off] condition (S_EB_(*f*_1/2_)). Note that the narrow shaded areas represent the SD across 10 trials, showing the robust and repeatable nature of the SSVEP paradigm. The grouping into peripheral and central channels is depicted at the bottom. The channels are consistently aligned on the *x*-axis, ***b***–***d***.

We presented flickering visual stimuli to awake and anesthetized flies while recording LFPs from different areas of the fly brain (Fig. 1*a–c*). Before characterizing the effects of anesthesia on the LFPs, we investigated whether different brain areas showed different responses to the visual flicker in 0% isoflurane (air). We first confirmed that in all our recordings (*N* = 16) we were able to detect the SSVEPs, whose magnitude depended on brain region and the location of the visual flicker (Fig. 2). When we presented 13 Hz flicker ipsilateral (on the same side) to the probe insertion site (Fig. 2*a*), we saw clear periodic waveforms in the time domain (Fig. 2*b*) as well as a clear peak at 13 Hz and its multiples (harmonics) in the frequency domain (Fig. 2*c*), reflecting robust SSVEPs. Figure 2*d* summarizes the average SSVEP power at 13 Hz (blue) and at its harmonic (26 Hz, red), for each channel. SSVEP power at both 13 Hz (*f*_1_) and 26 Hz (*f*_2_) was highest around channels 3–6, roughly corresponding to the medulla of the optic lobe (Fig. 1*c*, cyan structure), and lower responses in channels 8–14, corresponding to the higher-order central structures of the fly brain, as expected and consistent with other SSVEP studies in the fly (Paulk et al., 2013, 2015).

**Figure 3.**
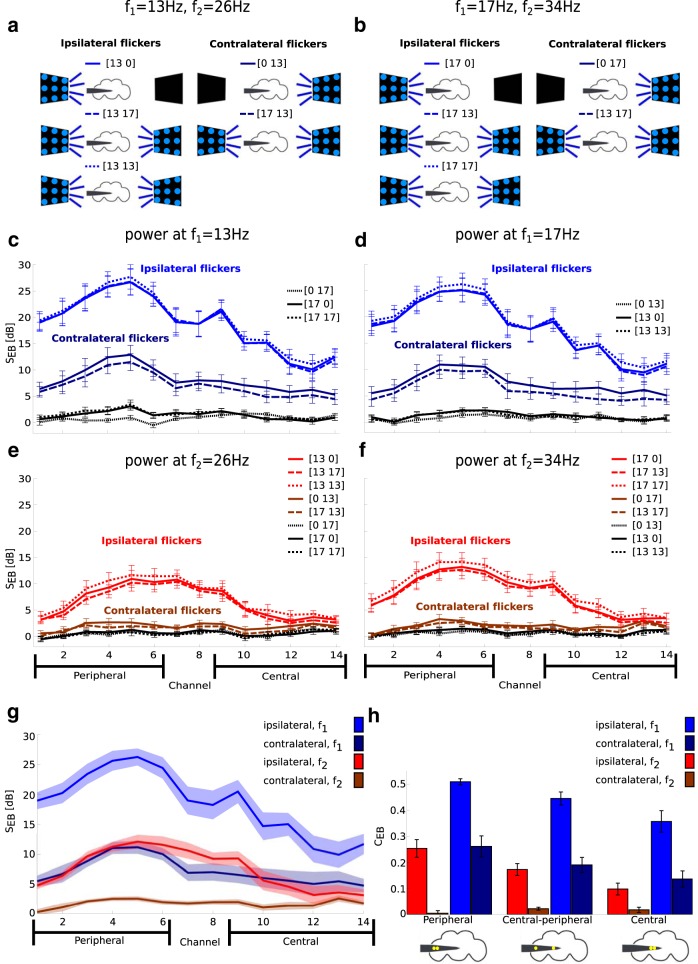
Baseline-corrected SSVEP power (S_EB_) and coherence (C_EB_) before anesthesia (0% isoflurane). ***a***, ***b***, Grouping trials according to ipsilateral and contralateral flicker configurations. A trial consisted of a presentation of one of the following eight flicker configurations: [off 13], [off 17], [13 off], [17 off], [13 13], [13 17], [17 13], and [17 17]. Trials were classified as either ipsilateral or contralateral according to the location of the flicker, with respect to electrode insertion site. ***a***, When analyzing SSVEP power at *f*_1_ = 13 Hz or *f*_2_ = 26 Hz, trials in which a single flicker was shown ([13 0] and [0 13], row 1) were classified according to the location of the flicker. Trials in which different flickers are shown at each panel ([13 17], [17 13], row 2) are classified according to the location of the 13 Hz flicker. Trials in which both panels show the same flicker ([13 13], row 3) are classified as ipsilateral, as this component dominated the response (see ***c–f***). Trials in which only a 17 Hz flicker is shown ([17 0], [0 17], and [17 17]) are excluded from the grouping. ***b***, Corresponding classification scheme when analyzing SSVEP power at *f*_1_ = 17 Hz or *f*_2_ = 34 Hz. ***c–f***, Group average (*N* = 13) baseline-corrected SSVEP power (S_EB_; see Local field potential analysis) for *f*_1_ = 13 (***c***), *f*_1_ = 17 (***d***), *f*_2_ = 26 (***e***), and *f*_2_ = 34 (***f***) Hz for each of the eight flicker configurations. Grouping flicker configurations as ipsilateral or contralateral accounted for much of the variance, as indicated by the color code (see legend). Error bars represent the SEM across flies (*N* = 13). ***g***, Group average (*N* = 13) baseline-corrected SSVEP power at *f*_1_ and *f*_2_ for ipsilateral and contralateral flicker configurations. Shaded area represents the SEM across flies. ***h***, Group average (*N* = 13) baseline-corrected SSVEP coherence (C_EB_) for P, CP, and C channel pairs. Schematics of the fly brain with superimposed examples of channel pairs from each grouping are shown at the bottom. SSVEP coherence followed a similar trend to SSVEP power: higher coherence at *f*_1_ than *f*_2_ and a decrease toward the center. Contralateral flickers evoked coherence predominantly at *f*_1_. Error bars represent SEM across flies (*N* = 13).

We observed clear and spatially specific responses for both frequency tags (13 and 17 Hz; main effect of channel location^a^, χ^2^ = 494.0, *p* < 10^−16^ See statistical table, row j–l for further detail.). However, we found that a subset of the eight flicker configurations could explain much of the variance in the SSVEP spatial response profiles we observed. Specifically, classifying flicker configurations as either ipsilateral or contralateral to the insertion site (Fig. 3*a*,*b*) allowed us to concisely summarize the SSVEP spatial response profiles in the first harmonic (*f*_1_ = 13 and *f*_1_ = 17; Fig. 3*c*,*d*, respectively) and second harmonic (*f*_2_ = 26 and *f*_2_ = 34; Fig. 3*e*,*f*, respectively) across all flicker configurations, into a single figure depicting the four combinations of harmonic and flicker location [(*f*_1_,*f*_2_) × (ipsilateral, contralateral); Fig. 3*g*]. SSVEP power for ipsilateral flicker configurations was much higher than contralateral ones (main effect of flicker location^b^: χ^2^ = 211.0, *p* < 10^−16^), which is in line with the visual information traveling from the optic lobes to the center of the brain (Fig. 3*g*; *N* = 13 flies, 0% isoflurane). For both ipsilateral and contralateral flickers, the responses were stronger at the first harmonic (*f*_1_ = 13 or 17 Hz) than at the second harmonic (*f*_2_ = 26 or 34 Hz; main effect of harmonic*^c^*: χ^2^ = 718.0, *p* < 10^−16^; Fig. 3*g*). In particular, SSVEP power for contralateral flickers at *f*_2_ was weakest and did not show increased responsiveness in peripheral channels (Fig. 3*g*; confirmed as the significant interaction between flicker location and harmonic^d^: χ^2^ = 190.0, *p* < 10^−16^). This observation suggests that the second harmonic, *f*_2_, reflects more local processing, which is evoked mostly when the flicker is presented to the ipsilateral side. In contrast, the first harmonic, *f*_1_, may reflect more global processing, showing a large response even when the flicker is presented to the opposite side of the insertion site.

In contrast to these large effects, separating the response profiles for ipsilateral and contralateral flicker configurations into the individual flicker configurations had a minimal but significant effect (main effect of flicker configuration^e^: χ^2^ = 17.6, *p* = 0.015), demonstrating that the grouping into ipsilateral and contralateral flicker configurations accounts for much of the variance.

### Evoked coherence varies across the fly brain

Next, we assessed whether SSVEP coherence showed brain region-specific patterns. Coherence measures the strength of linear dependency between two variables in the frequency domain (Bendat and Piersol, 2000), and was used in a similar preparation to investigate closed and open loop behavior in flies (Paulk et al., 2015). Paulk et al. (2015) observed increased SSVEP coherence when flies were engaged in closed-loop behavior, compared to open-loop behavior, where flies were not in control. Notably, SSVEP power alone did not distinguish between the two conditions.

To summarize the coherence data we grouped channels into periphery and center (Fig. 3*h*), and averaged channel pairs across the periphery, periphery-center, and center. Example pairs from each grouping are shown at the bottom of Figure 3*h*.

Overall, SSVEP coherence showed a pattern similar to that for SSVEP power. Higher coherence was observed at *f*_1_ (=13 or 17 Hz) than at *f*_2_ (=26 or 34 Hz), which is clearly seen by comparing blue (*f*_1_) and red (*f*_2_) bars in Figure 3*h* (main effect of harmonic^f^: χ^2^ = 179.0, *p* < 10^−16^), and at ipsilateral than at contralateral flickers, which are seen as lighter bars (ipsilateral) and darker bars (contralateral) in Figure 3*h* (main effect of flicker location^g^: χ^2^ = 29.5, *p* < 10^−6^). The effect of channel location is also strong, with the highest coherence observed between peripheral pairs, followed by peripheral-central pairs, and weakest for central pairs (Fig. 3*h*; main effect of channel location^h^: χ^2^ = 62.3, *p* < 10^−10^). Similarly to SSVEP power, there was an interaction between flicker location and harmonic^i^ (χ^2^ = 6.2, *p* < 0.02). While contralateral flicker configurations barely evoked coherent SSVEP activity throughout the brain at *f*_2_, they evoked location-dependent coherence at *f*_1_. Ipsilateral flicker configurations, however, evoked similar location-dependent coherence at both *f*_1_ and *f*_2_ (Fig. 3*h*). Together, these similar patterns of results for SSVEP power and SSVEP coherence (Fig. 3*g*,*h*) suggest a strong relationship.

The results so far have shown that SSVEP power and coherence have a characteristic spatial response profile, with higher values in the peripheral optic lobe than in the central regions, and that responses at *f*_2_ may reflect more local processing, as observed in the limited response to contralateral flicker configurations (Fig. 3*g*,*h*). In what follows, we investigated how a volatile general anesthetic, isoflurane, affects these distinct visual responses in the fly brain.

### Isoflurane reduces behavioral responses

**Figure 4.**
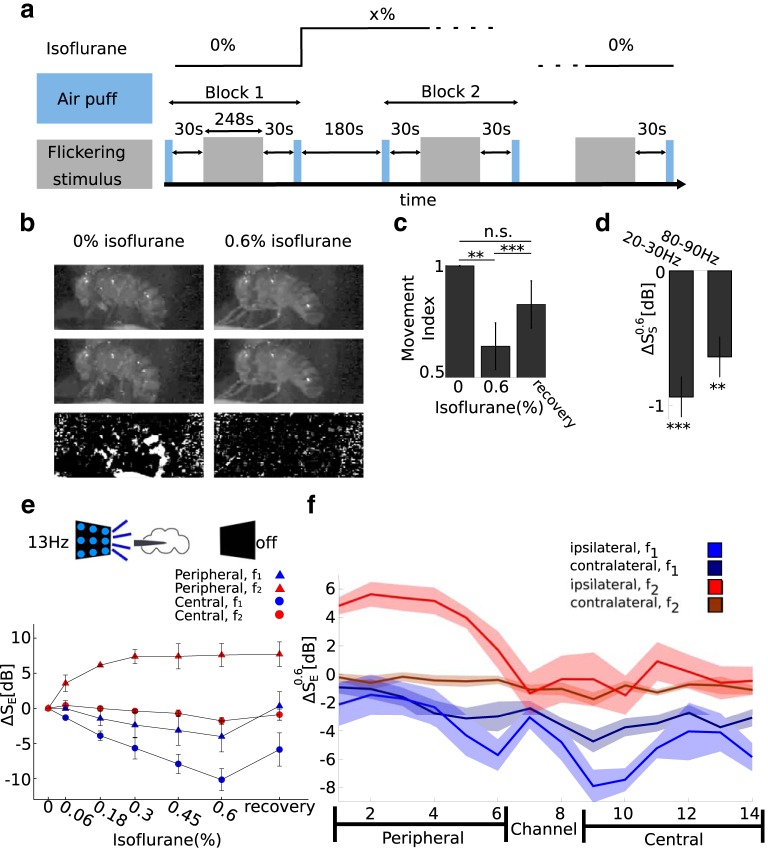
Isoflurane anesthesia has region- and harmonic-dependent effects on SSVEP power. ***a***, Experimental protocol. An experiment consisted of multiple blocks, each at a different concentration of isoflurane (top black line). Each block proceeded with (1) air puffs (light blue rectangles); (2) 30 s of rest; (3) 80 trials of flicker presentation, corresponding to 10 presentations for each of the eight flicker configurations (gray rectangles); (4) 30 s of rest; (5) air puff; (6) isoflurane concentration change; and (7) 180 s of rest for adjustment to the new isoflurane concentration. ***b***, Isoflurane abolishes behavioral responses. Consecutive video frames (first and second row) in response to an air puff before any anesthesia was administered (0%, left column) and before 0.6% isoflurane exposure (right column). In 0% isoflurane, flies respond to the air puff by moving, which is seen as the large difference in pixel intensity between consecutive frames (left, third row). After 0.6% isoflurane exposure, flies do not respond to the air puff, and there are only small differences between consecutive frames (right, third row). ***c***, Quantifying behavioral responses. Group average (*N* = 13) movement index (see Movement analysis) was reduced during exposure to 0.6% isoflurane and rebounded after isoflurane levels were reset to 0%. Error bars represent the SEM across flies. ***d***, Isoflurane reduces spontaneous brain activity (ΔS_s_), measured over four segments of 2.3 s before the start of the presentation of the visual stimuli. Group average (*N* = 13) effect of 0.6% isoflurane on spontaneous power (ΔS^0.6^_S_; see Local field potential analysis). Power is averaged across all channels. The average power for 20–30 and 80–90 Hz is significantly reduced. Error bars represent the SEM across flies. ***e***, Isoflurane reduces SSVEP power (ΔS_E_) at *f*_1_ but increases power at *f*_2_ in a concentration-dependent manner. SSVEP power at *f*_1_ = 13 Hz (blue) and *f*_2_ = 26 Hz (red) for the [13 off] flicker configuration (indicated by the schematic above), at increasing concentrations of isoflurane. For each fly, the SSVEP is first averaged over peripheral channels (triangles, channels 1–6) or central channels (circles, channels 9–14). The channel average is further averaged across flies (*N* = 3). Error bars reflect the SEM across flies. ***f***, Isoflurane increases SSVEP power at *f*_2_ for ipsilateral but not for contralateral flicker configurations. Spatial profile of SSVEP power at *f*_1_ (blue) and *f*_2_ (red) for contralateral (dark) and ipsilateral (light) flicker configurations in 0.6% isoflurane (ΔS^0.6^_E_). SSVEP power is averaged across ipsilateral or contralateral flicker configurations (Fig. 3*a*,*b*) first, then is averaged across flies (*N* = 13). Shaded areas represent the SEM across flies. The SSVEP power at *f*_1_ is reduced in central channels for all flicker configurations, indicating an effect on global neural processing. In contrast, SSVEP power at *f*_2_ is increased at the periphery, but only for ipsilateral flicker configurations, indicating an effect on local neural processing. The peripheral and central channels over which the average was taken in ***e*** are depicted at the bottom of ***f***. ***p < 0.001 and **p < 0.01 in ***c*** and ***d***.

In flies, like other animals, the behavioral effects of general anesthesia are investigated through behavioral responses to noxious stimuli, such as mechanical vibrations (Kottler et al., 2013; Zalucki et al., 2015a, Zalucki et al., 2015b). In our paradigm, we delivered a series of startling air puffs to the tethered fly exposed to different concentrations of isoflurane (Fig. 4*a*, blue rectangles). To quantify the responses to the air puffs, we analyzed video recordings of the experiments (see Movement analysis). Before any anesthesia (0% isoflurane), flies responded to the air puffs by moving their legs and abdomen, and this was visible as differences in pixel intensities between consecutive frames of the video recording (Fig. 4*b*, left column). The 0.6% isoflurane administration rendered flies completely inert, as was evident in the small differences between consecutive frames (Fig. 4*b*, right column). After the isoflurane concentration was reset to 0%, flies regained pre-anesthesia responsiveness (Fig. 4*c*): the MI (see Movement analysis) was significantly <1 at 0.6% isoflurane^j^ (*p* < 0.008, paired two-tailed *t* test; df = 12) and was not different from 1 at the end of the recovery period^k^ (*p* = 0.130). MI was significantly lower during 0.6% isoflurane administration than after the recovery period^l^ (*p* < 0.003). This analysis confirms that isoflurane abolishes behavioral responsiveness in fruit flies, as demonstrated in previous studies (van Swinderen, 2006; Kottler et al., 2013; Zalucki et al., 2015a, Zalucki et al., 2015b), and also that flies can recover from isoflurane in this preparation.

**Table 1:** Statistical table

	Data structure	Type of test	χ^2^/*t* score/ρ^2^	*p* value/confidence interval
aFixed effect: channel location	Normal distribution	Likelihood ratio test	χ^2^ = 494.0	*p* < 10^−16^
bFixed effect: flicker location	Normal distribution	Likelihood ratio test	χ^2^ = 211.0	*p* < 10^−16^
cFixed effect: harmonic	Normal distribution	Likelihood ratio test	χ^2^ = 718.0	*p* < 10^−16^
dInteraction: flicker location and harmonic	Normal distribution	Likelihood ratio test	χ^2^ = 190.0	*p* < 10^−16^
eFixed effect: flicker configuration	Normal distribution	Likelihood ratio test	χ^2^ = 17.6	*p* = 0.015
fFixed effect (coherence): harmonic	Normal distribution	Likelihood ratio test	χ^2^ = 179.0	*p* < 10^−16^
gFixed effect (coherence): flicker location	Normal distribution	Likelihood ratio test	χ^2^ = 29.5	*p* < 10^−6^
hFixed effect (coherence): channel location	Normal distribution	Likelihood ratio test	χ^2^ = 62.3	*p* < 10^−10^
iInteraction (coherence): flicker location and harmonic	Normal distribution	Likelihood ratio test	χ^2^ = 6.2	*p* < 0.02
jMI: 0% vs 0.6% isoflurane	Normal distribution	Two-tailed *t* test	*t* = 3.4	*p* < 0.008
kMI: 0% isoflurane vs recovery	Normal distribution	Two-tailed *t* test	*t* = 1.6	*p* = 0.130
lMI: 0.6% isoflurane vs recovery	Normal distribution	Two-tailed *t* test	*t* = -4.2	*p* < 0.003
mSpontaneous power 20-30 Hz: 0% vs 0.6% isoflurane	Normal distribution	Two-tailed *t* test	*t* = −6.25	*p* < 0.00005
nSpontaneous power 80-90Hz: 0% vs 0.6% isoflurane	Normal distribution	Two-tailed *t* test	*t* = −4.3	*p* < 0.002
oFixed effect: isoflurane	Normal distribution	Likelihood ratio test	χ^2^ = 631.36	*p* < 10^−16^
pInteraction: harmonic and isoflurane	Normal distribution	Likelihood ratio test	χ^2^ = 434.7	*p* < 10^−16^
qInteraction: Isoflurane and channel location	Normal distribution	Likelihood ratio test	χ^2^ = 187	*p* < 10^−16^
rInteraction: isoflurane and flicker location	Normal distribution	Likelihood ratio test	χ^2^ = 31.26	*p* < 0.01
sInteraction: isoflurane, channel location and harmonic	Normal distribution	Likelihood ratio test	χ^2^ = 23.09	*p* < 0.048
tSSVEP model fit: 0% isoflurane	Normal distribution	Confidence interval	ρ^2^ = 0.9	95% confidence interval for slope[1.0 1.21]; intercept [-6.01 -2.43]
uSSVEP model fit: highest dose of isoflurane	Normal distribution	Confidence interval	ρ^2^ = 0.95	slope = [1.05 1.15], intercept = [−1.70 0.75]
vSSVEP model fit: change between 0% and highest dose of isoflurane	Normal distribution	Confidence interval	ρ^2^ = 0.76	slope = [0.722 0.94], intercept = [2.50 4.48]
wFixed effect (coherence): isoflurane	Normal distribution	Likelihood ratio test	χ^2^ = 185	*p* < 10^−16^
xInteraction: harmonic and isoflurane	Normal distribution	Likelihood ratio test	χ^2^ = 146	*p* < 10^−16^
yInteraction: isoflurane and channel location	Normal distribution	Likelihood ratio test	χ^2^ = 29.25	*p* < 0.00001
zInteraction: isoflurane and flicker location	Normal distribution	Likelihood ratio test	χ^2^ = 12.35	*p* < 0.009
aaInteraction: isoflurane, harmonic and channel location	Normal distribution	Likelihood ratio test	χ^2^ = 0.66	*p* **=** 0.72
abCoherence model: $\Delta {\hat{\text{C}}}_{\text{FE}}$ vs $\Delta {\hat{\text{C}}}_{\text{E}}$	Normal distribution	Two-tailed *t* test	*t* = −5.47	*p* < 0.0001
avCoherence model: $\Delta {\hat{\text{C}}}_{\text{FE}}$vs$\Delta {\hat{\text{C}}}_{\text{FN}}$	Normal distribution	Two-tailed *t* test	*t* = −5.46	*p* < 0.0001
adCoherence model: $\Delta {\hat{\text{C}}}_{\text{FN}}$vs $\Delta {\hat{\text{C}}}_{\text{E}}$	Normal distribution	Two-tailed *t* test	*t* = −2.3	*p* < 0.040

### Isoflurane attenuates spontaneous brain activity

Previous work has shown that the attenuated motor behavior in fruit flies is accompanied by attenuated spontaneous brain activity, quantified as a reduction in mean power in the 20–30 and 80–90 Hz frequency bands (van Swinderen, 2006). We replicated the same effect here using the multiple electrode preparation, by averaging spontaneous power (= $S_{S}^{ik}(f)$; for the definition, see Local field potential analysis) across each frequency band (*f*) and across all channels (*i*) at *k* = 0.6% isoflurane concentration. Figure 4*d* shows the group average (*N* = 13) effect of 0.6% isoflurane on spontaneous power and confirms a significant reduction due to anesthesia in the 20–30^m^ and 80–90 Hz^n^ frequency bands (paired two-tailed *t* test: df = 12; *p* < 0.00005 and *p* < 0.002, respectively).

### Isoflurane has opposite effects on SSVEP power at *f*_1_ and *f*_2_

Previous studies in humans show that general anesthetics have spatially distinct effects on spectral power and coherence (Cimenser et al., 2011). In these studies, the primary sensory areas tend to remain reliably responsive, but higher-order areas show markedly reduce responsivity (Supp et al., 2011; Liu et al., 2012; Mashour, 2013). Isoflurane is known to potentiate GABAergic neurons, resulting in increased inhibition (Alkire et al., 2008; Garcia et al., 2010), and this is consistent with the attenuated spontaneous brain activity observed above and in previous reports (van Swinderen, 2006). Thus, we hypothesized that isoflurane would generally reduce neural responses, but that this reduction would be brain region dependent.

To assess the effects of anesthesia on SSVEP power, we presented visual flickers during exposure to increasing concentrations of isoflurane (Fig. 4*a*, gray rectangles).

In line with our expectations, we observed a concentration-dependent reduction in SSVEP power at *f*_1_. Furthermore, this reduction was more pronounced in central than peripheral areas (Fig. 4*e*, blue circles vs blue triangles; mean of channels 9–14, *N* = 3). Surprisingly, responses at *f*_2_ increased under anesthesia, but only in peripheral areas (Fig. 4*e*, red triangles vs circles). We confirmed a strong effect for isoflurane concentration (main effect of isoflurane^o^: *N* = 3, χ^2^ = 631.36, *p* < 10^−16^) as well as an interaction between harmonic and isoflurane^p^ (*N* = 3, χ^2^ = 434.7, *p* < 10^−16^).

To better understand the dissociation between the responses at *f*_1_ and *f*_2_, we collected data from 10 additional flies in which we manipulated isoflurane concentration in a binary manner [0% (air) → 0.6% → 0% (recovery)]. We found that isoflurane reduced SSVEP power at *f*_1_ (i.e., ${\text{ΔS}}_{\text{E}}\left(f_{1}\right)$ < 0) for both ipsilateral flicker configurations (Fig. 4*f*, light blue) and contralateral flicker configurations (Fig. 4*f*, dark blue). In contrast, isoflurane increased SSVEP power at *f*_2_ (i.e., ${\text{ΔS}}_{\text{E}}\left(f_{2}\right)$ > 0) for peripheral areas but only in response to ipsilateral flicker configurations (Fig. 4*f*, light and dark red). This region- and flicker-specific dissociation was confirmed by a strong interaction between isoflurane and channel location^q^ (χ^2^ = 187, *p* < 10^−16^) and isoflurane and flicker location^r^ (χ^2^ = 31.26, *p* < 0.01), as well as the triple interaction among isoflurane, channel location and harmonic^s^ (χ^2^ = 23.09, *p* < 0.048).

The reduction in SSVEP power at *f*_1_ was observed for both ipsilateral and contralateral conditions (Fig. 4*f*), suggesting a reduction in global levels of neuronal processing. Further, that this reduction is more pronounced in the central brain is consistent with isoflurane modulating sleep/wake pathways in the central brain (Kottler et al., 2013), in addition to possibly also impairing signal transmission from the periphery to the center. At the same time, the increase in SSVEP power at *f*_2_ in the periphery was not observed for contralateral flickers, suggesting that this increase may be attributed to isoflurane acting on some local circuit in the periphery. In the following, we provide a potential explanation with simple, yet biologically plausible, modeling of the SSVEPs.

### A minimal model explains the opposing effects of isoflurane on SSVEP power at *f*_1_ and *f*_2_

A global reduction in SSVEP power at *f*_1_ is in line with the reduced neural responsiveness described previously (van Swinderen, 2006) and with global impairment of neural communication across the brain (Alkire et al., 2008). However, the local increase in SSVEP power at *f*_2_ in the periphery is not consistent with these. Here, we propose a minimal model that explains these results in a quantitative manner.

First, we considered what type of processing of the input can result in a response at *f*_2_. Linear models of SSVEPs from human EEGs have demonstrated a reasonable fit to the observed data (Capilla et al., 2011). However, we can immediately reject purely linear models because our flickering stimuli consisted of a square wave, whose Fourier decomposition consists of only odd harmonics (*f*_1_, *f*_3_, *f*_5_,…). A linear transformation of the input signal cannot result in power at frequencies that are not present at the input in the first place (Norcia et al., 2015). This suggests that a nonlinear process is involved in the generation of the power at *f*_2_. The fly visual system is known to contain nonlinear processing (Egelhaaf and Borst, 1989; Reiff et al., 2010; Clark et al., 2011; Behnia et al., 2014)—could a physiologically based, well established nonlinearity account for the unexpected increase in power at *f*_2_ that we observed?

A prominent property in visual processing in animals, including fruit flies, is the segregation of the input pathway into luminance increment-responsive (On) and luminance decrement-responsive (Off) pathways (Joesch et al., 2010). The splitting of processing into these two pathways is captured by a nonlinearity in the form of half-wave rectification (Regan and Regan, 1988). Half-wave rectification is also implemented in the fly visual system (Reiff et al., 2010) and represents a biologically plausible, yet simple, nonlinearity.

**Figure 5.**
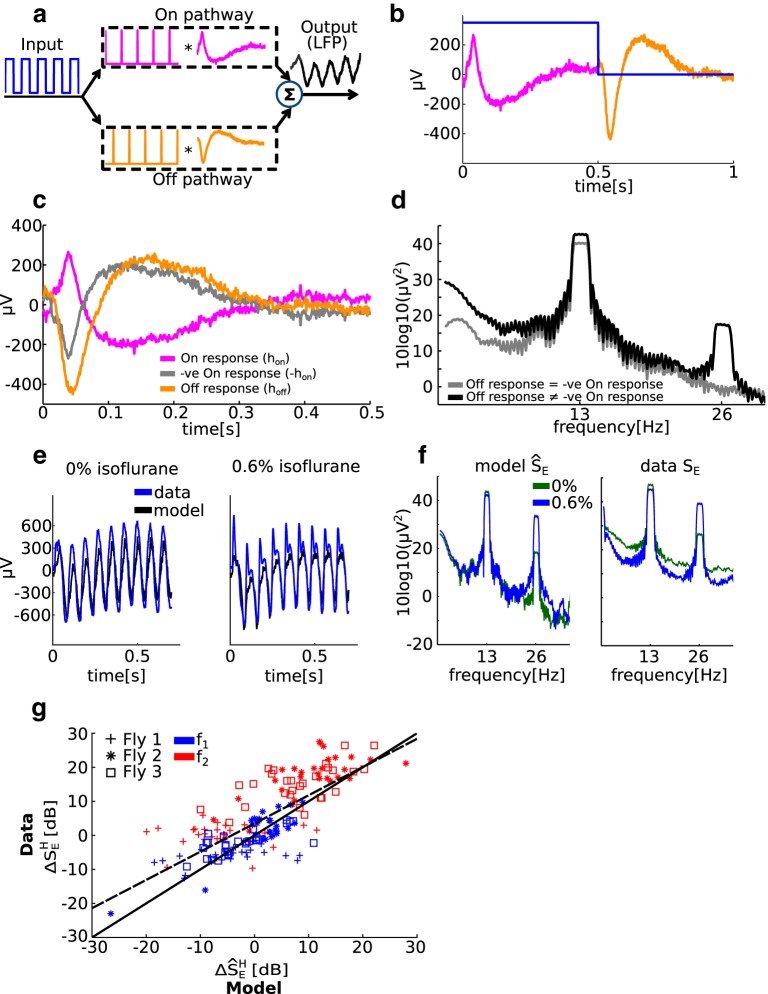
A minimal model explains the unexpected increase in SSVEP power at *f*_2_ due to isoflurane. ***a***, Modeling the SSVEPs. The input (depicted as a blue square wave) is (linearly) differentiated to extract points of luminance increments and decrements before splitting into two streams corresponding to the On (pink) and Off (orange) pathways. Each pathway is modeled as a linear operation determined by the impulse response of the pathway. The responses of the two pathways are summed to give the recorded SSVEP. ***b***, The impulse responses of the On and Off pathways were estimated from the response to a 1 Hz flickering stimulus (blue). An example from one channel is shown. No other parameters are fitted from the data. ***c***, Exemplar On (pink) and Off (orange) impulse responses obtained from the 1 Hz flicker presented in both panels [1 1] in 0% isoflurane (air). Note that the negative of the On impulse response (gray) is not identical to the Off impulse response. ***d***, The power spectra of the model output. When the negative of the On impulse response is the same as the Off impulse response, there is no power at *f*_2_ (gray). When the empirical On and Off impulse responses are used, the power spectrum has a sharp peak at *f*_2_ (black). ***e***, Comparison between the model output (black) and the recorded SSVEP (blue, average across 10 trials) to a [13 0] stimulus in 0% (left) and 0.6% isoflurane (right) in the time domain. An example from one channel is shown. ***f***, Corresponding comparison to ***e*** in the frequency domain. Spectra of model output (left) and recorded data (right, averaged across 10 trials of the [13 0] flicker configuration) in 0% (green) and 0.6% (blue) isoflurane show that the model correctly predicts that isoflurane increases SSVEP power at *f*_2_**_._*g***, The SSVEP model predictions are in excellent agreement with the observed effects of isoflurane. The model correctly predicts the reduction in power at *f*_1_ (blue) and the increase in power at *f*_2_ (red) for each of three flies (marked by a cross, square, or asterisk), across all channels (14) and both flicker configuration ([13 13] or [17 17]; *n* = 168, ρ = 0.76). The empirical line of best fit (dashed black) closely resembles the line of perfect fit (solid black).

Figure 5, *a* and *b*, summarizes our model, which is based on previous models of nonlinear SSVEP generation (Regan and Regan, 1988). First, the input is linearly differentiated to extract points of luminance change before passing through two opposite half-wave rectifiers, corresponding to segregation into the On and Off pathways. The result is two pulse trains with the same period as the input stimulus and a time delay of half of the stimulus period. The two pulse trains are separately linearly processed by the On and Off pathways, and are finally summed to give the recorded response. We estimated the impulse responses of the On and Off pathways for each channel from the response to a 20 s, 1 Hz flicker that was obtained before the main 13/17 Hz flicker blocks (Fig. 5*b*; see Modeling the SSVEPs).

The model predicts that if the Off pathway impulse response is the exact negative of the On pathway impulse response (Fig. 5*c*, gray line), there will be no power at the second harmonic (Fig. 5*d*, gray line). The symmetry between the On and Off responses cancels the nonlinearity (see Eq. 3.2 in Materials and Methods). When the impulse responses for the On and Off pathways are asymmetric, as is expected and consistent with known fly neurophysiology (Behnia et al., 2014), the half-wave rectification is in effect and a prominent peak at *f*_2_ is observed (Fig. 5*d*, black line).

Our minimal model is effective in explaining the opposing effects of anesthesia at *f*_1_ and *f*_2_ in the time (Fig. 5*e*) and frequency (Fig. 5*f*) domain representations of the SSVEP, explaining that isoflurane anesthesia increases the power at *f*_2_ by changing the impulse responses of the On and Off pathways. We emphasize that the model is completely determined by the response to the 1 Hz stimulus, which is then used to predict responses for the [13 13] and [17 17] flicker configurations. No parameters are fitted after computing the impulse responses.

To evaluate the model, we computed the correlation coefficient (ρ) and the line of best fit between the model-predicted and observed SSVEP power at *f*_1_ and *f*_2_ (see Evaluating the SSVEP model). We found excellent agreement between the model prediction and the actual data in 0% (air)^t^ isoflurane (*n* = 168; ρ^2^ = 0.9; 95% confidence interval: for slope, 1.0–1.21; for intercept, −6.01 to −2.43) and in the highest concentration of isoflurane delivered to each fly^u^ (*n* = 168; ρ^2^ = 0.95; 95% confidence interval: for slope, 1.05–1.15; for intercept, −1.70 to 0.75). Most importantly, the model accurately predicts the effects of isoflurane on SSVEP power. Figure 5*g* shows the observed effects versus the predicted effects of isoflurane on SSVEP power, and demonstrates that the model captures both the increase at *f*_2_ (red) and the decrease at *f*_1_ (blue) for each of three flies (marked by cross, asterisk, and square). The predicted and observed effects of isoflurane show a strong linear relationship^v^ (dashed black line; ρ^2^ = 0.76; df = 167; 95% confidence interval: for slope, 0.722–0.94; for intercept, 2.50–4.48) that closely resembles a perfect fit (solid black line).

Thus, assuming a minimal, yet biologically plausible, nonlinearity, our model provides a possible explanation for why isoflurane anesthesia unexpectedly increased SSVEP power at *f*_2_. Isoflurane is known to potentiate GABAergic neurons (Alkire et al., 2008; Garcia et al., 2010) so a “global” reduction in neural responses is expected. Consistent with this, we observed that SSVEP power at *f*_1_ was globally reduced, while SSVEP power at *f*_2_ increased, but only locally at the periphery. Our model, however, predicts that if isoflurane caused an imbalance between the On and Off pathways, the nonlinearity can be enhanced, causing increased SSVEP power at *f*_2_. The imbalance between the On and Off pathways is likely to emerge only when the local circuit is strongly engaged. Such strong engagement is much more likely for ipsilateral than for contralateral flicker configurations, which is consistent with the observation that the increase at *f*_2_ was observed only for ipsilateral flicker configurations. While our model cannot pinpoint the cellular/molecular mechanisms underlying this change, one potential cause is a widespread impairment in synaptic efficacy, independent of sleep circuits, that here results in affected On and Off responses (van Swinderen and Kottler, 2014; Zalucki et al., 2015).

### Isoflurane has opposite effects on SSVEP coherence at *f*_1_ and *f*_2_

We next investigated how isoflurane anesthesia affected the observed SSVEP coherence. Generally, the effects were closely related to the changes observed for SSVEP power. Following the same procedure for 0% isoflurane (Fig. 3*h*), we summarized the results by averaging coherence among pairs of recording sites within periphery, between periphery and center, and within center (see Analyzing SSVEP coherence). As expected, 0.6% isoflurane significantly modulated SSVEP coherence^w^ (main effect of isoflurane, χ^2^ = 185, *p* < 10^−16^).

**Figure 6.**
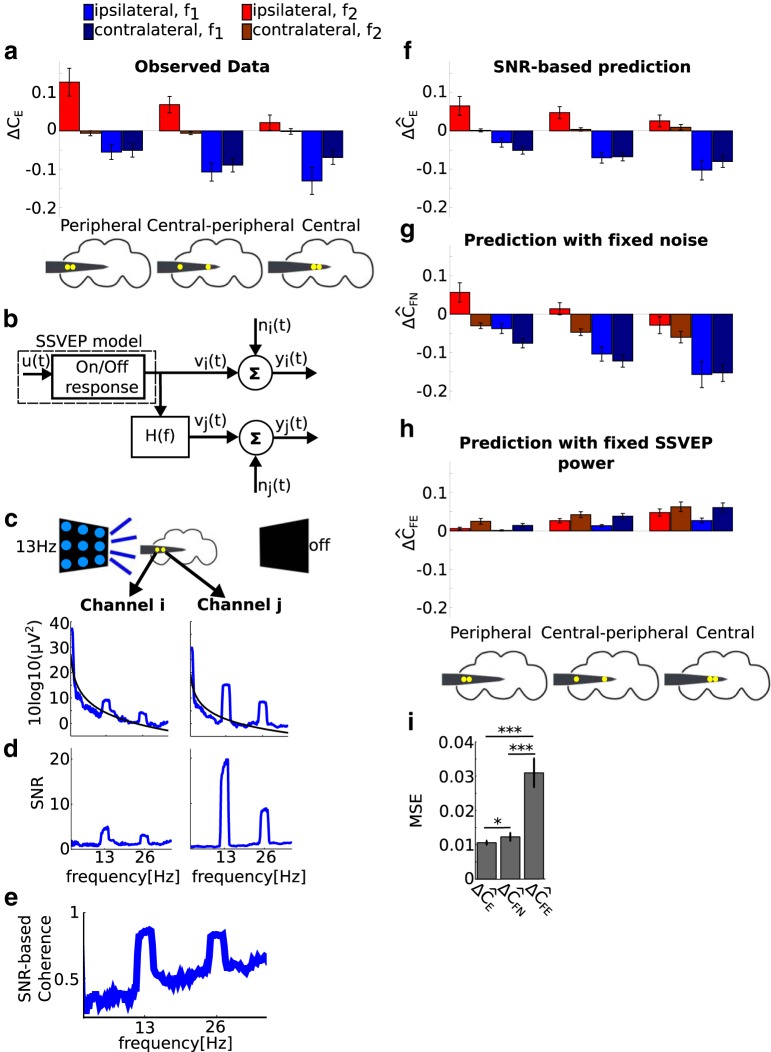
A minimal model based on the SNRs of SSVEP power explains the effects of isoflurane on SSVEP coherence. ***a***, Group average (*N* = 13) of the observed effects of anesthesia on SSVEP coherence (ΔC_E_). Isoflurane decreases SSVEP coherence at *f*_1_ (blue) and increases coherence at *f*_2_ (red). Isoflurane decreases coherence at *f*_1_ for all flicker configurations throughout the brain. Isoflurane increases SSVEP coherence at *f*_2_ at the periphery, but only for ipsilateral flicker configurations (light red). Schematics of the fly brain with superimposed examples of channel pairs from each grouping are shown at the bottom. Error bars represent the SEM across flies. ***b***, Linear framework for SNR-based coherence estimates. The SSVEP in channel *v_i_*(*t*) is related to the SSVEP in channel *v_j_*(*t*) through the transfer function *H*(*f*). Independent noise *n_i_*(*t*) and *n_j_*(*t*) enters at each channel separately to give the recorded SSVEPs *y_i_*(*t*) and *y_j_*(*t*). Under this scheme, SSVEP coherence has an analytic expression based on the SNR at each channel, given by Equation 4.1 (see SNR-based estimation of coherence). ***c–e***, Estimation of SNR and coherence prediction from the data. ***c***, Noise levels were estimated from nontagged frequencies at each channel, isoflurane concentration, and flicker configurations by fitting power-law noise to the SSVEP spectrum (see SNR-based estimation of coherence). Exemplar average SSVEP spectra and power-law fits for the [13 off] flicker configuration in 0% isoflurane for two channels, indexed by *i* and *j*, are shown. A schematic of the fly brain and channel locations is shown at the top. ***d***, The SNR of channels *i* and *j*, obtained by dividing the spectrum of the SSVEP by the power-law fit in the linear scale. ***e***, Example of SSVEP coherence prediction for channel *i* and *j* in ***d*** based on the SNR through Equation 4.1. ***f***, The SNR-based model correctly predicts the effects of isoflurane on coherence ($\Delta {\hat{\text{C}}}_{E}$). Group average (*N* = 13) SNR-based prediction of the effects of 0.6% isoflurane. The format and color scheme is the same as in ***a***. ***g***, ***h***, Coherence predictions using different definitions of the SNR. ***g***, Prediction based on SNRs using noise levels in 0% isoflurane ($\Delta {\hat{\text{C}}}_{FN}$; see SNR-based estimation of coherence). ***h***, Prediction based on SNR using SSVEP power levels in 0% isoflurane ($\Delta {\hat{\text{C}}}_{FE}$). ***i***, Quality of coherence prediction from each model. MSE between the observed (***a***) and each of the three predictions (***f–h***) averaged across all flies, channels, flicker configurations, and *f*_1_ and *f*_2_. This demonstrates that the effects of isoflurane on SSVEP coherence are largely attributed to the effects of isoflurane on SSVEP power, not on noise. Error bars represent the SEM across flies (*N* = 13). ***p < 0.001 and *p < 0.05.

The effects of 0.6% isoflurane on SSVEP coherence (ΔC*_E_*, *N* = 13 flies) are qualitatively similar to those on power, in terms of channel pair location, flicker location, and harmonic, as shown in Figure 6*a*. Isoflurane reduced coherence at *f*_1_ but increased coherence at *f*_2_ (Fig. 6*a*, red vs blue bars; interaction between harmonic and isoflurane^x^: χ^2^ = 146, *p* < 10^−16^). The reduction at *f*_1_ was greater in the center (Fig. 6*a*, right column), while the increase at *f*_2_ was predominantly observed at the periphery (Fig. 6*a*, left column; interaction between isoflurane and channel location^y^: χ^2^ = 29.25, *p* < 0.00001). The reduction at *f*_1_ was observed for all flicker configurations (Fig. 6*a*, light and dark blue), but the increase at *f*_2_ was only observed for ipsilateral flicker configurations^z^ (Fig. 6*a*, light red vs dark red; interaction between isoflurane and flicker location: χ^2^ = 12.35, *p* < 0.009). The triple interaction among isoflurane, harmonic, and channel was not significant^aa^ (χ^2^ = 0.66, *p* = 0.72). The results imply that in our paradigm there is a strong connection between SSVEP power and SSVEP coherence. In the following section, we dissect this by assuming a linear framework that provides an estimate of coherence based on the signal-to-noise ratios of tagged power in the frequency domain.

### A minimal model explains the opposing effects of isoflurane on SSVEP coherence at *f*_1_ and *f*_2_

The observation that isoflurane affected coherence and power in a similar way suggests that in our data the two measures are linked. We explain the opposing effects of isoflurane on SSVEP coherence at *f*_1_ and *f*_2_ with another simple model. The model assumes that in each pair of channels, one channel (Fig. 6*b*, *v_i_*(*t*)) receives an input from the initial sensory processing (i.e., Fig. 6*b*, On/Off response box) and the other (*v_j_*(*t*)) is a linearly filtered version of the first (represented by the transfer function *H*(*f*)). Finally, independent noise (*n_i_*(*t*) and *n_j_*(*t*)) enters at each channel, giving the two output voltages (*y_i_*(*t*) and *y_j_*(*t*)). This simple framework allows us to apply an analytic derivation of coherence based on the SNRs at each channel (see Signal-to-noise ratio-based estimation of coherence; Bendat and Piersol, 2000).

The model involves two main assumptions about the nature of the LFP. First, the relationship between the SSVEPs in each pair of channels is linear. Second, spontaneous activity is independent of evoked activity. There is evidence that purely linear processing can provide a good description of the neural responses in the *Drosophila* brain (Bialek et al., 1991; Behnia et al., 2014), and independence between evoked and spontaneous neural activity is often assumed when assessing neural connectivity in human LFP studies (Truccolo et al., 2002; Wang et al., 2008). Thus, both assumptions are physiologically plausible. However, given the present lack of understanding regarding the physiological underpinning and inter-area properties of the LFP, we cannot propose a mechanistic, physiology-based perspective for this model.

To quantify the SNRs, we first estimated the noise level by fitting power-law noise to the power spectrum at the nontagged frequencies during visual stimulation for each channel, flicker configuration, and isoflurane concentration (Fig. 6*c*; see SNR-based estimation of coherence). Note that the SSVEP paradigm allows us to operationally regard power at the tagged frequency (*f*_1_ and *f*_2_) as signal and power at non-tagged frequencies as noise (Norcia et al., 2015). Dividing the measured SSVEP power by the estimated noise levels (in the linear scale) provides our estimation of the SNR (Fig. 6*d*; see SNR-based estimation of coherence). The SNR estimates together with Equation 4.1 provide a coherence estimate (Fig. 6*e*). Finally, we separately obtained estimates of the SSVEP coherence in 0% and 0.6% isoflurane concentrations to predict the effects of isoflurane on coherence (${\Delta \hat{\text{C}}}_{\text{E}}$; Fig. 6*f–h*).

The predicted effects of isoflurane on SSVEP coherence is in excellent agreement with the observed data (Fig. 6*a*, observed coherence, *f*, for the model prediction). The model captures the general decrease of coherence at *f*_1_ as well as the increase of coherence in the periphery at *f*_2_ for ipsilateral flicker configurations.

In this framework, the effects of isoflurane on noise level as well as on SSVEP power both contribute to the SNR-based prediction of coherence. But what is the relative contribution of nontagged noise and tagged signal to our successful prediction of SSVEP coherence? To isolate the relative contribution, we recalculated the SNR by fixing either noise or signal to 0% isoflurane levels, which we call SNR_FN_ and SNR_FE_ (see Separating the contribution of “noise” and “signal” to the SNR-based estimation of coherence). The results (Fig. 6*g*,*h*), clearly show that the contribution of the signal (or evoked response) is much more important for the model prediction.

We formally confirmed the above observation by computing the mean squared error between each SNR-based prediction ($\Delta {\hat{\text{C}}}_{\text{E}}$, $\Delta {\hat{\text{C}}}_{\text{FN}}$, and $\Delta {\hat{\text{C}}}_{\text{FE}}$) and the observed data ($\Delta {\text{C}}_{\text{E}}$) across all channel pairs and flicker configurations (Fig. 6*i*; see Separating the contribution of noise and signal to the SNR-based estimation of coherence). Disregarding the effect of isoflurane on power ($\Delta {\hat{\text{C}}}_{\text{FE}}$) resulted in considerably worse predictions than^ab^ $\Delta {\hat{\text{C}}}_{\text{E}}$ (*p* < 0.0001, df = 12) and ^ac^ $\Delta {\hat{\text{C}}}_{\text{FN}}$ (*p* < 0.0001, df = 12). However, disregarding the effects of isoflurane on noise resulted in only slightly (but significantly) worse predictions^ad^ ($\Delta {\hat{\text{C}}}_{\text{E}}$ vs $\Delta {\hat{\text{C}}}_{\text{FN}}$: *p* < 0.040, df = 12). This means that the observed effects of isoflurane on SSVEP coherence, which is a global decrease of coherence at *f*_1_ and a local (peripheral) increase of coherence at *f*_2_, is largely attributed to the effect of isoflurane on SSVEP power at the tagging frequency of the stimulus, rather than to general effects on nontagged frequencies.

## Discussion

In this study, we showed that isoflurane has distinct local and global effects on the fruit fly brain. This was made possible by our approach that combines pharmacological manipulation of the states of the brain through anesthetics, perturbation of the neural circuits through periodic visual stimuli, and analysis and modeling of behavior and neural data. Together, these components synergistically provide a fuller picture of the effects of isoflurane anesthesia on visual processing, which may generalize to the brains of animals other than flies.

As to the mechanisms of anesthesia, recent studies (Alkire et al., 2008; Mashour, 2013) suggest that reduced cortical communication is at the core of the anesthetic-induced loss of consciousness. In particular, increased synchronous activity induced by anesthesia has been suggested to adversely interfere with the communication between brain areas (Supp et al., 2011; Lewis et al., 2012; Sarasso et al., 2014), which may explain the failure of the propagation of evoked responses from primary to higher-order areas (Supp et al., 2011; Liu et al., 2012; Mashour, 2013). The volatile general anesthetic isoflurane also abolishes behavioral responses in fruit flies at concentrations similar to those required for human anesthesia (van Swinderen, 2006; Kottler et al., 2013; Zalucki et al., 2015a, Zalucki et al., 2015b; present study), suggesting that the neural mechanisms through which this anesthetic works may be conserved in most animals. Here we questioned whether isoflurane has distinct effects on local and global processing in the fruit fly brain, and thereby investigate whether an entirely different brain neuroanatomy might reflect similar fundamental effects on neural processing under general anesthesia.

Using a multielectrode preparation allowed us to record from different brain areas simultaneously, and to assess brain region-dependent effects. By presenting flickering visual stimuli, we could isolate the neural response in the frequency domain. The frequency decomposition revealed specific effects of anesthesia on the first harmonic (*f*_1_, 13 or 17 Hz) and second harmonic (*f*_2_, 26 or 34 Hz), which reflected global and local visual processing. Our results show that the reduction in behavioral responses is accompanied by attenuated spontaneous brain activity, and this was also true for the SSVEPs in the central brain, which were reduced for all stimulus configurations, indicating an effect on global neuronal processing at *f*_1_. In contrast, and to our surprise, local responses at *f*_2_ in peripheral areas increased, but only for ipsilateral flicker configurations. Modeling the SSVEPs was crucial to understand this unexpected effect, explaining that the *f*_2_ power increase in the periphery can be attributed to isoflurane-induced changes of the On and Off response pathways in the optic lobes. We further showed that the analogous effects of isoflurane on coherence can be explained by explicitly considering how isoflurane affects the tagged brain activity (both *f*_1_ and *f*_2_). Overall, the reduction in SSVEP power and coherence in the central brain fits with the view that general anesthetics target interarea neural communication, impairing the transmission of the visually evoked responses from the optic lobes to central brain structures.

One possibility to describe our finding is to separate the effects of isoflurane into the central and the peripheral, rather than the local and global, as we presented the effects in the article. We think that using the term “central effect” is not as well suited because it fails to capture the fact that the effect of isoflurane on SSVEP power and coherence at *f*_1_ generalized over flicker configurations (both ipsilateral and contralateral; Fig. 4*f*). In addition, we argue that this effect involves the transmission of the signal between the periphery and the center. As such, we think that the response at *f*_1_ reflects global processing, rather than central processing, and *f*_2_ reflects local (which can be also considered as peripheral) processing.

### Evoked and spontaneous activity

The characterization of evoked responses, as opposed to spontaneous activity, through the delivery of a controlled input can reveal additional information about the system. In our experiment, the SSVEPs increased in peripheral areas at *f*_2_ for specific flicker configurations, revealing a clear difference between the effects of isoflurane on the periphery and center of the fly brain. The use of evoked activity in studying general anesthesia may be particularly important because it allows tracking a stimulus-related neural process across the brain, potentially making it easier to identify impaired interarea communication. In SSVEP paradigms, the signal is operationally defined as activity at the tag and its harmonics, and this assumption makes it straightforward to define SNRs of evoked activity. This is more difficult with spontaneous activity, where signal and noise cannot be easily separated. Our operational definition of signal and noise, following the tradition of SSVEP studies (Norcia et al., 2015), allowed us to explicitly consider how SSVEPs at the tag frequency combine with nontagged activity (through the quantification of the SNR) to influence coherence. In our data, the effects of isoflurane on SSVEP coherence could be largely attributed to the effects of isoflurane on SSVEP power, as opposed to effects on surrounding, non-stimulus-related activity (Fig. 6*f–i*).

Focusing on neural activity at predefined frequencies, however, is also a limitation of SSVEP paradigms as this only probes the behavior of the system in a narrow range: the tag and its harmonics. This is particularly important in the context of nonlinear systems whose frequency response can be highly input dependent. We expect that both our modeling of the SSVEPs and the SNR**-**based estimation of coherence will need to be expanded when the system is evaluated over a broader dynamic range.

### Neural substrate of the SSVEPs

Our modeling of the SSVEPs concisely yet plausibly accounts for the unexpected increase in power at *f*_2_ observed in the periphery. Given the vast literature on elementary motion detection circuitry in flies (Egelhaaf and Borst, 1989; Reisenman et al., 2003; Borst and Euler, 2011), it may be possible to provide more comprehensive modeling. However, for the purpose of explaining the unexpected effects of anesthesia, our minimal modeling was sufficient and provided a physiologically plausible explanation: isoflurane most likely affected the responses of local On and Off pathways, which, combined with the presentation of a periodic stimulus, resulted in increased power at *f*_2_. We note that, in principle, the model can be used to predict the response to arbitrary flicker configuration, but in this work we focused on the observation that required further explanation: the unexpected increase in power at *f*_2_. We are currently investigating whether isoflurane-resistant mutants, which have recently been identified (Kottler et al., 2013; Zalucki et al., 2015), can be used to further clarify the mechanisms involved in the global decrease and local increase in responsiveness that we observed.

Even for our simple model, it is not straightforward to assign a fine neural substrate to the SSVEP because there are many connections between the fly optic lobes, such that stimulation in one lobe causes activation in the other (Haag and Borst, 2008). While our recordings (and those by Paulk et al., 2015) clearly show that SSVEP power is much smaller when the flicker is presented to the opposite eye, the broad-field flicker prevents us from precisely disentangling the relative contribution of each optic lobe to the LFP. Another factor is the aggregate nature of the LFP; while the first On and Off responsive cells may be observed as early as the lamina (Reiff et al., 2010), we cannot tell how much these cells contribute to the LFP, compared to other downstream neurons. Future studies separating the contributions of the On or Off pathways to the LFP via genetic manipulations and the use of stimuli that target each pathway separately will help to clarify the neural substrate of the SSVEP.

### Slow-wave and interarea neural communication

Sleep and general anesthesia are defined by similar criteria, and there is evidence for some shared mechanisms (Franks, 2008). The involvement of sleep mechanisms in the impairment of cortical communication observed in general anesthesia (Ferrarelli et al., 2010; Sarasso et al., 2015) is not established, but one possibility is that the stereotypical DOWN states that manifest as the human EEG slow wave and are observed in both general anesthesia and non-REM sleep states, may prevent long-range coordinated activation (Sarasso et al., 2014).

Recent findings extend the proposed relationship between sleep and anesthesia to fruit flies, in which genetic manipulations of sleep circuits can confer both resistance and hypersensitivity to isoflurane (Kottler et al., 2013). However, to date, there has been no evidence of a slow wave in sleep or anesthesia in flies (van Swinderen, 2006; Kirszenblat and van Swinderen, 2015), and we found no evidence of it here. Thus, anesthetics may target sleep circuits in all brains but only produce a slow wave in some. Instead, the mechanism for the reduced responsiveness in the central brain that we observed under isoflurane may be a combination of potentiated sleep circuits and compromised synaptic efficacy, which has been demonstrated in flies (van Swinderen and Kottler, 2014; Zalucki et al., 2015). While sleep circuits seem unlikely to modulate the responses of the peripheral On and Off pathways, the globally compromised synaptic efficacy could cause an imbalance in the responses of the On and Off pathways, resulting in the unexpected increase in power at *f*_2_.

### Outlook

Bottom-up approaches that focus on molecular mechanisms have considerably improved our understanding of anesthetic drugs and have identified a promising set of potential target sites (Franks, 2008; Garcia et al., 2010; Brown et al., 2011). On the other hand, it remains unclear how effects at the molecular level affect large-scale neuronal circuits. Instead, top-down approaches that focus on global effects are providing evidence that general anesthetics share a common end point in the reduction of interarea communication (Lee et al., 2013; Mashour, 2014; Sarasso et al., 2015). Using the metrics developed for characterizing these global effects (Casali et al., 2013; Lee et al., 2015) in conjunction with the genetic manipulations available in *Drosophila* is a promising direction. Studies that manipulate the state of the brain and external perturbations can be combined with signal-processing techniques and modeling to help us understand how anesthetic effects at the molecular level change the global state of the brain.

## Synthesis

The decision was a result of the Reviewing Editor Gustavo Deco and the peer reviewers coming together and discussing their recommendations until a consensus was reached. A fact-based synthesis statement explaining their decision and outlining what is needed to prepare a revision is listed below. The following reviewers agreed to reveal their identity: Karin Nordström, Miguel Dasilva

Review 1:

The first para in the Introduction is too vague to be useful. For example, the sentence on lines 41-44, does not really say anything, but is then continued with "..to reconcile these varied observations..." Which varied observations are you referring to?

In general in the introduction it is very unclear what species you are referring to. I assume that the second paragraph refers to humans, but this is never specified. Since you are comparing flies with humans, it is paramount that you specify who you are talking about.

I am not convinced about your grouping of experiments into ipsilateral and contralateral, described in Figure 3a, as the stimuli are quite different (no stimuli on half the screen, vs different stimuli). The grouping may be perfectly sound, but needs to be justified, with data.

The main point (based on the title) of the paper is described on page 24, i.e. after many pages of reading. I think it is a bit of a shame that it takes so long to get to this, and that many busy readers may thus miss it. Is there any way to simplify figures 1-2 and the associated text (as these are mostly methods figures), so that readers do not give up before getting to the results?

Lines 552, 553 and 554 have superscript letters after 'period'. What do these mean?

The paragraph on lines 558-575 seems to belong under the next heading.

On line 624 you introduce biological non-linearities. All fly vision people are drilled in the knowledge that the visual system is full of non-linearities, so you might want to rephrase this not to annoy this audience.

Again on line 647, it is well established that the ON and OFF pathways are asymmetric (See any recent paper from the Reiser, Clandinin or Borst lab). If you do not want to annoy people from the fly vision community, rephrase.

Lines 671-674, your reasoning here is not very clear. Please clarify.

Additionally I do not understand why the f1 harmonic is so large in the periphery for contralateral stimulation (Figure 3b and 3c)? Could that be an effect of your definition into contralateral and ipsilateral, or how do you think this signal has traveled from the contralateral visual field to the medulla on the other side?

Figure 2a: blue on black does not print well at all. Can you modify?

Figure 2c, please include a scale bar for the y-axis.

Can you make figure 4b bigger, as it is an important control (you can make figure 4f smaller).

Review 2:

In my opining there are some concerns that should be explained by the authors:

1. The classification of flicker configurations as ipsilateral or contralateral to the insertion site seems controversial and would need further explanation. It would be most helpful to see that ipsilateral and bilateral stimulations at the same frequency do not show significant differences. In that regard, further explanation would be required on how trials where different tagging frequencies were used at both sites were classified as ipsi or contralateral. Proof of their similarity with those trials where one single frequency was presented at both sites would also be needed.

2. The authors claim that the second harmonic reflects more local processing, because f2 is stronger when the flicker is shown to the ipsilateral site. However, according to their classification of the different types of flickers, both ipsilateral-only and bilateral-at-the-same-frequency are included in this category. This, at least, should be discussed. Also, why do f2-ipsilateral and f1-contralateral (Figure 3b) have similar power?

3. Is the Chi-square test the best way to show significant differences in all cases? What I mean is that it is difficult to follow whether statistical significance accounts for one specific frequency or both. For example, in figure 3c, why is an ANOVA not used to show the differences between central and peripheral locations? Please, explain.

4. In terms of the minimal model to explain the effects of isoflurane on SSVEP power, all presented data refer to ipsilateral stimulation, could the model account for contralateral stimulation too? Did the authors consider testing experimentally the prediction that isoflurane changed the balance of the ON, OFF responses? Why not? This would need some explanation.

5. What is the reason for applying the minimal model based on SNRs? Can the authors justify from a physiology-related perspective the necessity of using this model in the same way they did for the previous one related to SSVEP power?

6. The authors claim that they distinguish between local and global effects of isoflurane. I wonder whether it would be more precise talking about central and peripheral effects. Could the authors, please justify the use of the former term?

7. In the discussion (line 824), the authors admit that "the broad-field flicker prevents us from precisely disentangling the relative contribution of each optic lobe to the LFP" Could the authors explain whether this represents a concern when establishing the difference between ipsi and contralateral stimulation in terms of the scope of the paper?
